# Comprehensive Plasma Metabolomic Profile of Patients with Advanced Neuroendocrine Tumors (NETs). Diagnostic and Biological Relevance

**DOI:** 10.3390/cancers13112634

**Published:** 2021-05-27

**Authors:** Beatriz Soldevilla, Angeles López-López, Alberto Lens-Pardo, Carlos Carretero-Puche, Angeles Lopez-Gonzalvez, Anna La Salvia, Beatriz Gil-Calderon, Maria C. Riesco-Martinez, Paula Espinosa-Olarte, Jacinto Sarmentero, Beatriz Rubio-Cuesta, Raúl Rincón, Coral Barbas, Rocio Garcia-Carbonero

**Affiliations:** 1 Clinical and Translational Oncology Laboratory, Gastrointestinal Unit, i+12 Research Institute Hospital 12 de Octubre, 28041 Madrid, Spain; bsoldevilla.imas12@h12o.es (B.S.); alens.imas12@h12o.es (A.L.-P.); ccarretero.imas12@h12o.es (C.C.-P.); alasalvi@ucm.es (A.L.S.); bgil.imas12@h12o.es (B.G.-C.); criesco@salud.madrid.org (M.C.R.-M.); espinosa_pau@gva.es (P.E.-O.); jacinsarmentero@yahoo.es (J.S.); beatriz230794@hotmail.com (B.R.-C.); rrincon.imas12@h12o.es (R.R.); 2 Spanish National Cancer Research Center (CNIO), 28029 Madrid, Spain; 3 Centre for Metabolomics and Bioanalysis (CEMBIO), Department of Chemistry and Biochemistry, Facultad de Farmacia, Universidad San Pablo-CEU, CEU Universities Urbanización Montepríncipe, Boadilla del Monte, 28660 Madrid, Spain; ang.lopez.ce@ceindo.ceu.es (A.L.-L.); alopgon@ceu.es (A.L.-G.); cbarbas@ceu.es (C.B.); 4 Oncology Department, Hospital Universitario 12 de Octubre, 28041 Madrid, Spain; 5 Department of Medicine, Faculty of Medicine, Complutense University of Madrid (UCM), 28040 Madrid, Spain

**Keywords:** NETs, disease modelling, machine learning, metabolic signaling, molecular pathways, plasma metabolites, diagnostic biomarkers

## Abstract

**Simple Summary:**

Metabolic flexibility is one of the key hallmarks of cancer and metabolites are the final products of this adaptation, reflecting the aberrant changes of tumors. However, the metabolic plasticity of each cancer type is still unknown, and specifically to date, there are no data on metabolic profile in neuroendocrine tumors. The aim of our retrospective study was to assess the metabolomic profile of NET patients to understand metabolic deregulation in these tumors and identify novel biomarkers with clinical potential. We provided, for the first time, a comprehensive metabolic profile of NET patients and identifies a distinctive metabolic signature in plasma of potential clinical use, selecting a reduced set of metabolites of high diagnostic accuracy. We have identified 32 novel enriched metabolic pathways in NETs related with the TCA cycle, and with arginine, pyruvate or glutathione metabolism, which have distinct implications in oncogenesis and may open innovative avenues of clinical research.

**Abstract:**

Purpose: High-throughput “-omic” technologies have enabled the detailed analysis of metabolic networks in several cancers, but NETs have not been explored to date. We aim to assess the metabolomic profile of NET patients to understand metabolic deregulation in these tumors and identify novel biomarkers with clinical potential. Methods: Plasma samples from 77 NETs and 68 controls were profiled by GC−MS, CE−MS and LC−MS untargeted metabolomics. OPLS-DA was performed to evaluate metabolomic differences. Related pathways were explored using Metaboanalyst 4.0. Finally, ROC and OPLS-DA analyses were performed to select metabolites with biomarker potential. Results: We identified 155 differential compounds between NETs and controls. We have detected an increase of bile acids, sugars, oxidized lipids and oxidized products from arachidonic acid and a decrease of carnitine levels in NETs. MPA/MSEA identified 32 enriched metabolic pathways in NETs related with the TCA cycle and amino acid metabolism. Finally, OPLS-DA and ROC analysis revealed 48 metabolites with diagnostic potential. Conclusions: This study provides, for the first time, a comprehensive metabolic profile of NET patients and identifies a distinctive metabolic signature in plasma of potential clinical use. A reduced set of metabolites of high diagnostic accuracy has been identified. Additionally, new enriched metabolic pathways annotated may open innovative avenues of clinical research.

## 1. Introduction

Reprogrammed metabolism encompasses the capacity of cells to respond or adapt their metabolic signaling to support and enable cell survival in unfavorable or hostile conditions. This ability is enhanced in cancer cells in order to improve their adaptive phenotype and maintain both viability and uncontrolled proliferation. Metabolic flexibility is therefore one of the key hallmarks of cancer [1], although the pathways involved in the metabolic plasticity of each cancer type remain to be elucidated. Metabolites are the final products of this adaptation, reflecting the aberrant changes in the genomic, transcriptomic and proteomic variability of tumors, and provide therefore useful biological and clinical information on cancer initiation and progression [2,3,4]. This, together with the fact that metabolomics can be easily performed in readily accessible biological samples (plasma, urine), makes metabolic profiling of cancer patients a promising tool to characterize the tumor phenotype and identify novel biomarkers of potential clinical use. Systems medicine approaches integrating high-throughput “-omic” technologies into diagnostic platforms have indeed enabled the detailed analysis of metabolic networks (known as metabolomics) in several cancers of high incidence, prevalence and mortality [5,6,7,8], but these do not include neuroendocrine neoplasms (NENs).

NENs comprise a heterogeneous family of rare tumors of increasing incidence and challenging clinical management [9]. Although they can arise in virtually any organ, the most common primary tumor sites are the lungs (25%) and the digestive tract (~65%). Well-differentiated neuroendocrine tumors (NETs) account for ~80% of all NENs, have a rather indolent clinical behavior, as compared to their exocrine counterparts, and are associated with a good to moderate prognosis depending on primary tumor site, proliferative index (ki67 or mitotic index) and tumor stage. About 20% of NETs have also the unique ability to produce and secrete amines or peptide hormones to the blood stream, the so-called “functioning tumors”, that produce specific endocrine syndromes (i.e., carcinoid syndrome) that may seriously impair patients quality of life and prognosis [10]. Survival has improved over time for all NETs, likely reflecting earlier diagnosis and improvements in therapy [11,12]. However, a significant proportion of patients are still diagnosed with advanced stages of disease, highlighting the need to identify novel specific biomarkers that may contribute to an earlier detection and an increased likelihood of cure. 

The study of hereditary genetic syndromes associated with an increased predisposition to develop NETs (~5%) has contributed to partially elucidate some of the mechanisms involved in their tumorigenesis [13,14,15,16,17,18]. Germline mutations in MEN1, RET, CDKN1B, VHL, NF1 and TSC1/2 are the molecular alterations most frequently detected in hereditary NENs. Although some of these mutations have a relevant representation in sporadic NETs (i.e., MEN1, TSC1/2), other molecular alterations involved in epigenetic regulation, DNA repair, telomeres regulation and chromosomal rearrangements have been also implicated [19,20,21]. Despite these recent advances, however, the molecular mechanisms of NET genesis and progression remain largely unraveled. In addition, few authors have explored NETs from a metabolomic perspective. A pilot prospective study analyzed urine samples from 28 gastroenteropancreatic NET patients by nuclear magnetic resonance (NMR) spectroscopy, and showed distinct metabolomic phenotypes by primary tumor site (small bowel versus pancreatic NEN) and function [22]. A second recent work described the metabolomic fingerprint of 46 small intestine NET tissues analyzed by NMR spectroscopy, suggesting the existence of complex metabolic pathways in NETs, possibly influencing tumor development and evolution, and thereby clinical outcome [23]. With the exception of these two small studies, the metabolomic profile of patients with NETs has not been studied to date.

In this context, the aim of our study was to perform a comprehensive metabolic profiling of NETs to better understand metabolic dysregulation in these tumors and identify novel biomarkers of potential clinical use. To this aim, multiplatform untargeted metabolomic analyses were performed in plasma samples of 77 patients with advanced gastrointestinal and lung NETs, and 68 non-cancer individuals (controls). The diagnostic potential and biological relevance of differential metabolites identified was assessed, and dysregulated pathways were explored to provide further insight into the molecular mechanisms involved in NET development and progression.

## 2. Results 

### 2.1. Metabolomic Profiling in Plasma of Patients with Neuroendocrine Tumors 

The metabolite fingerprint was assessed using a multiplatform LC-MS, GC-MS and CE-MS approach in plasma of 77 patients diagnosed with NETs and of 68 non-cancer individuals (controls). Main characteristics of the study population are summarized in Table S1. All patients had well-differentiated G1-2 NETs (33.8% G1 and 66.2% G2), the most common primary tumor site was the small intestine (58.4%) and one-third had functioning tumors (carcinoid syndrome). 

Data obtained after peak alignment and filtering were used for multivariate analysis of unsupervised principal components (PCAs) to verify the distribution of QCs in each technique. System stability, performance and reproducibility of sample treatment procedures were reflected with the spontaneous grouping of these QC samples (Figure S1).

**Figure 1:**
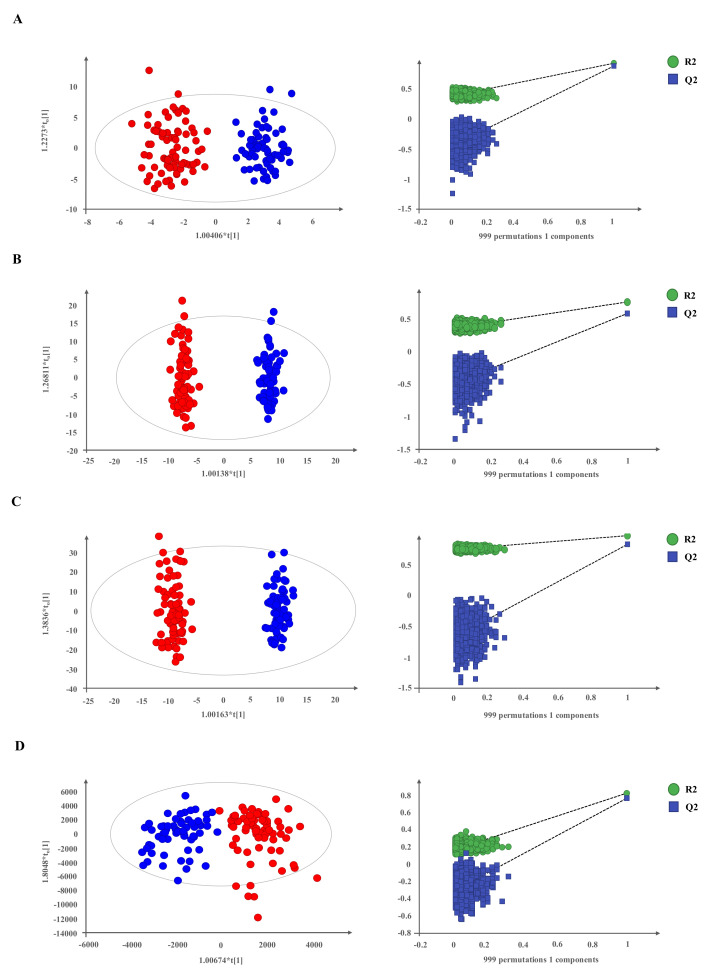
Supervised models show a clear separation between NET patients and non-cancer individuals. (**A**–**D**) OPLS-DA score plots and permutation tests of OPLS-DA models for each platform through 999 permutations. Panel (**A**) for CE-MS data (R2 = 0.872, Q2 = 0.843); panel (**B**), LC-MS/ESI(+) data (R2 = 0.954, Q2 = 0.871); panel (**C**), LC-MS/ESI(−) data (R2 = 0.885, Q2 = 0.788) and panel (**D**), GC-MS data (R2 = 0.781, Q2 = 0.744). Red dots, NETs (*n* = 77); blue dots, non-cancer individuals (*n* = 68).

For each platform in multivariate analysis, unsupervised (Figure S2), and supervised PLS-DA and OPLS-DA models were also conducted. OPLS-DA supervised models were used to model differences between groups and were validated using permutation tests (Figure 1A–D). 

All analytical techniques clearly discriminated NET patients from non-cancer individuals in the applied models. A total of 1006 metabolites were detected and univariate analysis revealed the following individually significant differential metabolites between cases and controls: 75 compounds in CE–MS, 150 in LC–MS ESI(+), 296 in LC–MS ESI(−) and 19 in GC–MS. These variables were annotated and/or identified as described in “Annotation and compound identification” in the Material and Methods section and are summarized in Table 1. 

The integration of metabolic data acquired by different analytical platforms resulted in 155 identified metabolites with a differential availability in NET patients (*p* < 0.05), when compared to non-cancer individuals. Metabolite identification of some specific metabolites (arginine, glutamine, phenylalanine, among others) across more than one analytical platform significantly increases the confidence of metabolite identification (Table 1). No significant differences were found by gender, age, grade, primary tumor site and hormonal syndrome. 

### 2.2. NETs Show a Particular Signature of Metabolites with Diagnostic Potential

The unsupervised heatmap cluster plot of the 155 metabolites identified with a differential availability in NET patients as compared to non-cancer individuals is shown in Figure S3. Given the relevant capacity to discriminate NET patients from non-cancer individuals, we applied ROC analysis and calculated the AUC of each identified metabolite to determine their individual performance as NET diagnostic biomarkers. We also calculated the variable importance in projection (VIP) score from the OPLS-DA model. VIP score estimates the importance of each metabolite in the model and therefore, their ability to discriminate NETs from non-cancer patients. Table S2 summarizes the ROC and OPLS-DA analyses of the 155 differential plasmatic metabolites identified between NETs and controls. Those with an AUC > 0.85, a VIP > 1.0 and a |*p*(corr)| > 0.5 or both were considered as metabolites with biomarker potential. Twenty-seven metabolites had both an AUC > 0.85 and a VIP > 1.0 and a |*p*(corr)| > 0.5; 17 metabolites had only a VIP > 1.0 and a |*p*(corr)| > 0.5 and 5 metabolites had only an AUC > 0.85. Overall, thus, we identified 49 metabolites with significant diagnostic potential.

**Figure 2:**
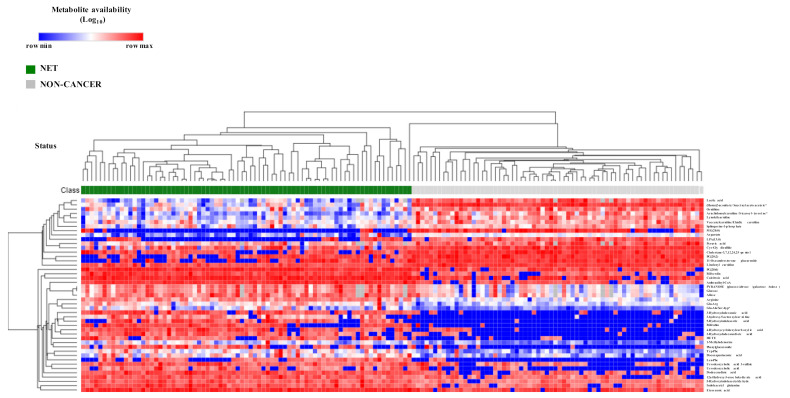
Metabolite biomarker candidates show high diagnostic potential of NET patients. Unsupervised hierarchical heatmap of differential plasmatic metabolites (*n* = 48) between NET (*n* = 77) and non-cancer (*n* = 68) patients. All samples are shown in columns and metabolites in rows. Hierarchical clustering was performed on rows and columns using One minus Pearson correlation metric and average as linkage method. Individual values were coded as colors, ranging from blue (row minimum) to red (row maximum). This analysis clearly discriminated two clusters, one encompassing all NET patients and the other one all non-oncologic control patients.

Next, to determine which molecules were directly related to NETs independent of other clinical factors, we performed a logistic regression model (LRM) for each selected metabolite adjusted for gender, age, glycemia, creatinine levels and selected concomitant drugs as potential confounding variables before the inclusion in the model (Table S3). Only up to two significant drug associations (*p* < 0.05) were included in the LRM of each metabolite in order to avoid overfitting due to excessive explicative features. Out of the 49 metabolites assessed, only one metabolite, suberyl-glycine, was not significantly contributing to explain the classification output in its model (Table S4), indicating a poorer diagnostic ability. Thus, 48 metabolites were significantly contributing to explain the classification model (NET vs. non-cancer patients), independent of other confounding factors. The unsupervised heatmap cluster plot of these 48 metabolites clearly discriminated two clusters, one gathered all NET patients and the other one all the non-cancer individuals (Figure 2).

Finally, to validate the identity, differential expression and biomarker potential of these 48 metabolites, we performed a targeted metabolomic analysis (LC- QQQ-MS) of 13 selected metabolites based on their nature, stability and ability to be analyzed and quantified (Table S5), as described in Material and Methods.

**Figure 3:**
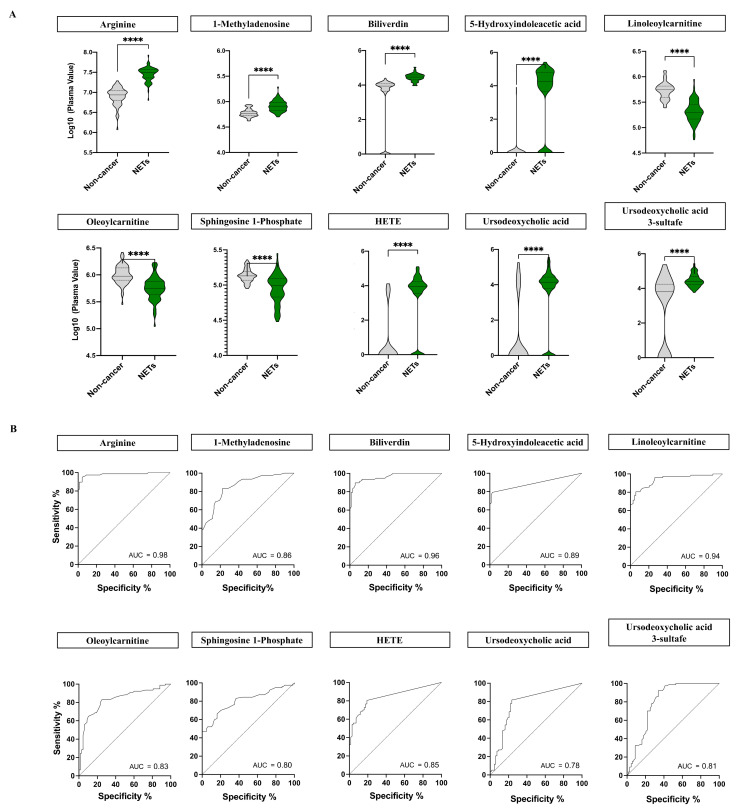
(**A**) Plasma abundance of the 10 validated metabolites with diagnostic potential in NETs. Violin plots and non-parametric Mann-Whitney U test were performed to assess the abundance and distribution of the 10 validated metabolites in NET patients (*n* = 77) and non-cancer individuals (*n* = 68). Log plasma values in NET patients are shown in green whereas non-cancer individuals are plotted in grey. Continuous lines correspond to the median values whereas dashed lines relate to quartiles Overall, all the validated metabolites showed significant differences (****; *p* < 0.0001) between NET and non-cancer individuals. (**B**) Receiver operating characteristic (ROC) curves of the 10 validated metabolites with biomarker potential in NETs. The curves were built based on the area under the curve (AUC) analysis of patients (*n* = 77) and non-cancer individuals (*n* = 68). The optimal cut-off points were selected according to the maximization of the Youden Index. Overall, all the validated metabolites showed high biomarker potential with AUC > 0.75.

Arginine, 1-methyladenosine, biliverdin, 5-hidroxyindolacetic acid, linoleoylcarnitine, oleoylcarnitine, sphingosine-1-phosphate, 15-hidroxyeicosatetraenoic acid, ursodeoxycholic acid and ursodeoxycholic acid 3-sulfate identities were confirmed as potential diagnostic biomarkers of NETs, while bilirubin, 3-hidroxydodecanodioic acid and 3-hidroxydodecanoic were not (Table S6), probably due to their instability. Distribution of plasma abundance and ROC curves of the validated metabolites with diagnostic potential in NETs and non-cancer patients were showed in Figure 3.

### 2.3. Biochemical and Functional Nature of Identified Metabolites in NET Patients

**Figure 4:**
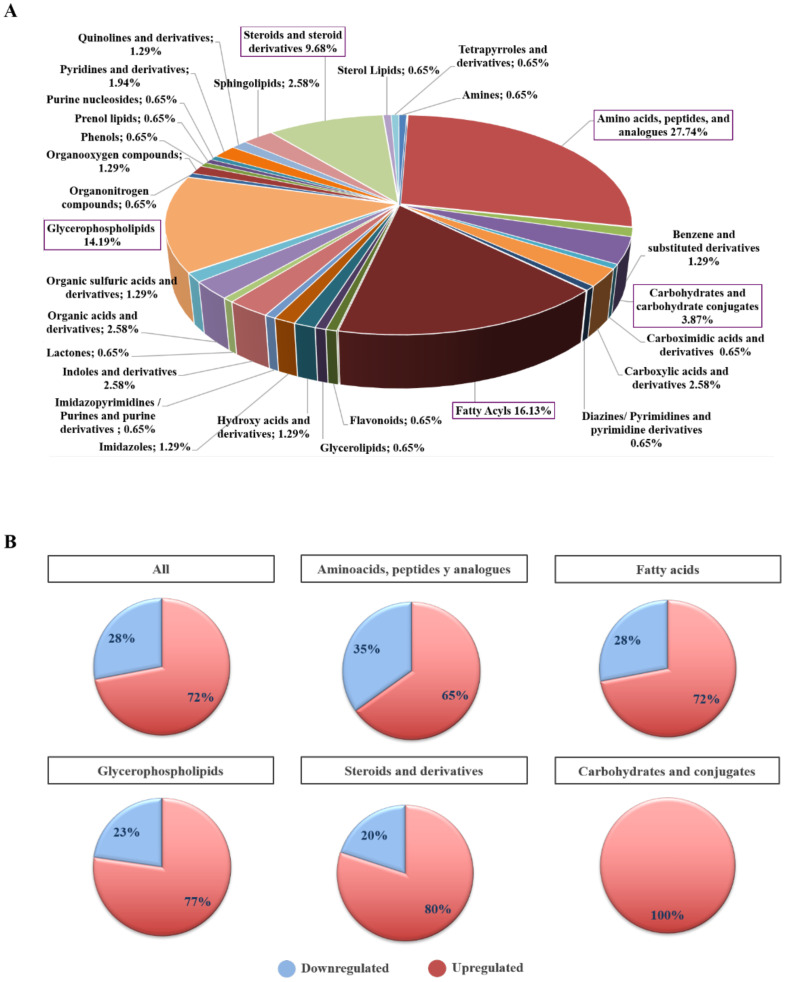
Biochemical classification of differential plasma metabolites in NET patients. (**A**) Pie chart showing the percentage distribution of biochemical classes of differential and annotated plasmatic metabolites (*n* = 155) in NETs (*n* = 77) vs. non-cancer (*n* = 68) individuals. Metabolites were classified into 28 classes according to their biochemical nature. This analysis revealed amino acids, peptides and analogues (*n* = 43; 27.74%), fatty acyls (*n* = 26; 16.13%), glycerophospholipids (*n* = 22; 14.19%), steroids and steroid derivatives (*n* = 16; 9.68%), and carbohydrates and carbohydrate conjugates (*n* = 6; 3.87%) as the most represented biochemical classes. (**B**) Bar chart summarizing the percentage of up- and downregulated plasmatic metabolites in the main biochemical classes found to be biologically relevant in NET (*n* = 77) vs. non-cancer (*n* = 68) individuals. Overall, upregulation (72%) prevailed over downregulation (28%) in our metabolite set (*n* =155). This upregulated vs. downregulated metabolite trend remained constant for all main biochemical classes: amino acids, peptides and analogues (65% vs. 35%), fatty acyls (72% vs. 28%), glycerophospholipids (77% vs. 23%), steroids and steroid derivatives (80% vs. 20%), and carbohydrates and carbohydrate conjugates (100%).

To better understand the molecular nature of metabolites involved in NETs we classified the 155 differential metabolites according to their biochemical class. The main predominant categories identified were amino acids, peptides and their derivatives (27.7%), fatty acids (16.1%), glycerophopholipids (14.2%), steroids and derivatives (9.7%) and carbohydrates and their conjugates (3.9%) (Figure 4A). Seventy-two percent of all differential metabolites were upregulated in NET patients. The proportion of up- or downregulated metabolites by biochemical class is summarized in Figure 4B.

Interestingly, the main predominant categories of lipids following the official classification [24] were glycerophospholipids, fatty acids and sterols. Glycerophospholipids represent the largest class of lipids found to be altered. Lyso forms were increased in contrast to glycerophosphocolines and glycerophosphoethanolamines which were downregulated. Oxidized lysoglycerophospholipids (oxLPCs) were found with increased abundances in the NET group, and there are very few studies to date where oxLPCs have been measured. Fatty acids and their derivatives account for about 30% of the measured lipids, and it is easily noticed that there was an overall decrease in carnitine levels. In addition to that, we have been able to identify increased levels of oxidized derivatives of arachidonic acid (HETE) [25]. Other predominant group, sterols, consisted mainly of bile acids and had a high content in the NET group.

NET patients also presented higher levels of sugars and citric acid and lower levels of lactic and pyruvic acid, which are key metabolites in glycolysis and the tricarboxylic acid cycle (TCA)). A remarkable increase in serotonin and its principal metabolite, 5-hydroxyindoleacetic acid, was also detected in the NET cohort, a hormone secreted in excess in patients with carcinoid syndrome.

### 2.4. Differential Metabolites in NET Patients Are Related with Molecular Pathways Associated with Cancer

Considering the functional relevance of the selected metabolites documented in the literature, we found several enriched pathways related to oncogenesis, some specifically involved in NET development, such as angiogenesis, mTOR pathway, tryptophan metabolism, Warburg effect, oxidative stress, and urea cycle, among others (Figure S5A). The proportion of metabolites up- or downregulated by the pathway is summarized in Figure S5B.

**Figure 5:**
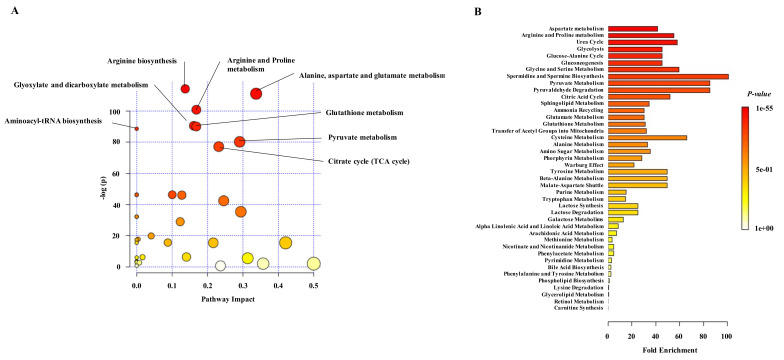
Pathway analysis of differential plasma metabolites in NET patients. (**A**) Metabolite Pathway Analysis (MPA) representing the significant enriched pathways (FDR < 0.05) by availability of selected metabolites (*n* = 155) in plasma of NET patients. The x-axis indicates the impact of matched metabolites of our dataset on the pathway from the topology analysis. The -log(pval) is plotted in the y axis and shows to which extent the pathway is enriched. Circle size represents the impact factor of matched metabolites in the pathway, and circle color indicates the pathway enrichment significance. The most enriched pathways among the 32 significant ones were: arginine biosynthesis (FDR: 1.0143 × 10^−48^); alanine, aspartate and glutamate metabolism (FDR: 2.2363 × 10^−47^); arginine and proline metabolism (FDR: 6.3629 × 10^−43^); glyoxylate and dicarboxylate metabolism (FDR: 1.8621 × 10^−38^); glutathione metabolism (FDR: 2.8086 × 10^−38^); aminoacyl-tRNA biosynthesis (FDR: 1.1784 × 10^−37^); pyruvate metabolism (FDR: 5.0802 × 10^−34^) and citrate cycle (FDR: 1.0634 × 10^−32^). (**B**) MSEA of differential plasma metabolites in NET patients. *X*-axis represents the fold enrichment of each metabolite set and the colour of the bars indicates the raw *p*-value. Thirty-four metabolite sets were significantly enriched (FDR < 0.05). Aspartate metabolism (Q = 28.809), arginine and proline metabolism (Q = 38.272), urea cycle (Q = 40.438), glycolysis (Q = 31.619) and glucose and alanine cycle (Q = 31.619) were the most significantly enriched metabolite sets.

Pathway analysis of the 155 differential metabolites identified several molecular dysregulated pathways in NETs. Metabolite Pathway Analysis (MPA) showed that arginine biosynthesis, arginine and proline metabolism, animoacyl-tRNA biosynthesis, citrate cycle and pyruvate, glutathione, glyoxylate, dicarboxylate, alanine, aspartate and glutamate metabolisms were the most commonly dysregulated pathways in NET patients (Figure 5A and Table S7). Moreover, Metabolite Set Enrichment Analysis (MSEA), where we used different sets of metabolites from MPA, suggested that tryptophan metabolism and urea cycle, among others, were also dysregulated in NETs (Figure 5B and Table S8).

MPA and MSEA performed with the 48 metabolites with greater diagnostic potential confirmed arginine biosynthesis, arginine and proline metabolism, amimoacyl-tRNA biosynthesis, citrate cycle, pyruvate and glutathione metabolism, and urea cycle were the most relevant dysregulated pathways in NET patients (Figure S4, Tables S9 and S10).

## 3. Discussion

Our study provides, for the first time, a comprehensive metabolomic profile of NETs, assessed by multiplatform untargeted metabolomic profiling of plasma samples in a large cohort of patients with advanced disease, and identifies a distinctive metabolic signature of potential clinical use. The integration of metabolic data acquired by GC, CE and LC coupled to MS identified 155 differential compounds between NETs and non-cancer patients. ROC and OPLS-DA analysis revealed 49 specific metabolites of diagnostic potential, 48 of which significantly contributed to the model after adjustment for other potential confounding variables such as gender, age, glycemia, creatinine levels and concomitant drug therapy. The unsupervised heatmap cluster plot of these 48 metabolites clearly identified two distinct clusters, one encompassing all NET patients and the other one all non-cancer individuals. Although biochemical assessment of several peptides is currently used in the clinic for the diagnosis and follow-up of NET patients, the use of general tumor markers such as chromogranin A (CgA) or neuron-specific enolase (NSE) is not recommended for screening nor are they sufficiently reliable as sole diagnostic procedures as they may be increased in several other oncological and non-oncological conditions [26]. In this context, the high diagnostic accuracy of the identified metabolites in our study may provide very valuable new tools to improve the specific detection of NETs.

Differential metabolites identified were related with classical cancer pathways (apoptosis, cell cycle) and NET signaling (tryptophan metabolism, angiogenesis or the mTOR pathway). In addition, we identified 32 novel enriched metabolic pathways in NETs related with the TCA cycle, and with arginine, pyruvate or glutathione metabolism, which have distinct implications in oncogenesis. To date, only two small studies have partially explored the metabolomic profile of NET patients, but none of them using plasma samples as main source of metabolic analysis. Kinross et al. conducted a prospective pilot study that analyzed urine samples of 28 gastroenteropancreatic NETs by 1H-NMR spectroscopic profiling. Distinct metabolomic phenotypes were identified by primary tumor site (small bowel versus pancreatic NEN) and function, and they also observed that variations in hippurate metabolism strongly contributed to the class description. This study had, however, important limitations such as the limited sample size, the substantial age gap between control and tumor populations, and the lack of control of other potential confounding variables such as gender, renal function or concomitant drug therapy [22]. More recently, Imperiale et al. assessed the metabolomic fingerprint of 46 small intestinal NET primary tumors and 18 liver NET metastases by 1H-NMR spectroscopy, as compared to 30 normal small intestine and liver samples, and results suggested alterations in crucial metabolic pathways such as the tricarboxylic acid cycle (TCA cycle). Our study also shows an increase in the TCA cycle activity, reflected by the high availability of isocitrate/citrate compounds in the plasma of NET patients (+64%). Moreover, high levels of glucose (+174%), glutamine (+69%) and fatty acids, which fuel the TCA cycle further support the hypothesis of TCA upregulation in NETs. Studies conducted in other hormone-dependent tumors, such as prostate cancer, emphasize the relevance of altered intermediary metabolism in malignant transformation. More specifically, the metabolic transformation of citrate-producing normal cells to citrate-oxidizing malignant cells has been implicated in oncogenesis. Citrate oxidation in the TCA cycle to produce ATP has important implications on cellular bioenergetics, cell growth, apoptosis, lipogenesis and angiogenesis [27]. The TCA cycle is a convergence point in the cellular respiration machinery, strictly regulated to fulfill cell bioenergetics, biosynthetic and redox balance requirements. Although several tumors types are characterized by a marked deregulation of TCA enzymes [28], its involvement in cancer metabolism remains incompletely understood.

The presence of high levels of isocitrate/citrate in plasma of NET patients could also derive from exported citrate from the mitochondrial pool to be used for lipogenesis [29]. Our data show a characteristic lipidome in NET patients, mainly represented by the enrichment of glycerophospholipids, fatty acids and sterols. More specifically, oxidized lysoglycerophospholipids (oxLPCs) were found with increased abundances in NETs indicating a strong oxidative stress in these tumors, as well as high levels of oxidized derivatives of arachidonic acid (HETE), and a major decrease in carnitine levels [25]. L-carnitine is an essential metabolite, critical for the bidirectional transport of long-chain fatty acyl and the acyl coenzyme A between the cytosol and the mitochondria, which has been considered a bottleneck in the metabolism control of cancer cells [30,31]. Recent reports suggest that the carnitine system is essential for the metabolic adaptation of cancer cells, which obtain energy from beta-oxidation of lipids. Thus, low levels of carnitine in plasma of NET patients may be related to the active carnitine system in tumor mitochondria and the upregulation of beta-oxidation pathways.

Arachidonic acid (AA) is a polyunsaturated fatty acid, which is subsequently metabolized through three different enzymatic pathways (cyclooxygenase (COX), lipoxygenase (LOX) and cytochrome (CYP) P450) leading to a wide variety of lipid mediators (HETE) involved in multiple physiological and pathophysiological processes [32]. In addition to high levels of AA in plasma of NET patients, we have also found an important increase of eicosanoids derivatives, intimately related to inflammatory responses [32].

Tumor progression is also dependent on cholesterol metabolism as proliferating cells increase cholesterol uptake. Cancer cells adapt the high requirements of intracellular cholesterol through different mechanisms including the endogenous production of cholesterol and fatty acids, a reduction of their efflux through transporters or an increase in the uptake of low-density lipid particles [33]. Plasma of NET patients show high content of sterols, mainly bile acids, and cholesterol derivatives such as vitamin D, biliverdin and bilirubin, suggesting the dependency of NETs on lipid and sterol metabolism.

Vascularization and angiogenesis have a particular relevance in NET development and progression [34], and several differential metabolites identified in our study may contribute to the angiogenesis switch. For example, arginine was found to be upregulated in NETs and is the main source of nitric oxide. NO exhibits both anti- and pro-tumoral effects, and is deeply involved in the regulation of angiogenesis, apoptosis, cell cycle, invasion and metastasis [35]. Similarly, lysophosphatidic acid, HETEs or biliverdin induce angiogenesis by upregulation of VEGFA, VEGFC, IL-1β and IL-8 [36,37,38]. Overall, these findings further support the relevant role that angiogenesis plays in the pathogenesis of NETs.

The mTOR pathway is also critical in NETs [39]. In fact, an mTOR inhibitor, everolimus, has demonstrated antiproliferative activity in these neoplasms and is approved for the treatment of advanced gastrointestinal, pancreatic and lung NETs [40,41]. In our study, we detected very high plasma levels of arginine (+243%) and glutamine (+69%) in NET patients, which are, together with leucine, stimulators of mTOR via the regulation complex. Moreover, an increased abundance of phosphatidylcholine (+51% PC(32:0), +39% PC(38:2) and −28% PC(38:5)) was also observed, the synthesis of which is promoted by mTORC1. Interestingly, the mTOR pathway has been associated with cancer through its role in the regulation of polyamine dynamics [42]. Polyamine levels are associated with a reduction of apoptosis and an increase of cancer cell proliferation and expression of metastasis-related genes, although the mechanisms underlying these effects have not been well defined [43]. Of note, we detected a 38% increase in the acetylspermidine polyamine, illustrating the relevance of polyamines metabolism inNETs. Recently, Chalishazar et al. observed that MYC-driven small-cell lung cancer (SCLC) preferentially depends on arginine-regulated pathways, including polyamine biosynthesis and mTOR pathway activation [44]. ASS1, which indirectly produces arginine in the urea cycle, is often decreased or even abolished through epigenetic silencing in many cancers, including SCLC. Moreover, ASS1 knockdown results in increased mTOR activity and in arginine auxotrophy. Thus, arginine deprivation could be a promising therapeutic strategy for cancers that depend on arginine for their survival [3]. Finally, we also found abundance differences in hypoxanthine. Low levels of hypoxanthine both in urine and plasma samples are often observed in cancer patients, especially in patients with advanced disease stages [45], as hyperproliferative tissues require increased DNA synthesis. Accordingly, we observed a 34% decrease of hypoxanthine abundance in the plasma of NET patients. The underlying mechanism of hypoxanthine downregulation in NET patients is unclear, but it is plausible that alterations in purine metabolism may occur during tumor progression. Consistent with this hypothesis, an inverse correlation was found in our study between hypoxanthine plasma levels and the tumor proliferative rate or Ki-67 index (r2 = −0.243, *p* = 0.033).

One of the strengths of our study is that it was performed in plasma samples of a large and homogeneous population, uniformly and prospectively collected and analyzed. Moreover, a set of metabolites was validated in a target analysis with a different analytical platform in the same cohort. Nevertheless, results shall be further investigated in an independent NET patient cohort and metabolomic profiling of patients with exocrine tumors of similar tissue origin (lung and gastrointestinal carcinomas) would be very helpful to validate the specific metabolomic profile of NETs and to confirm the diagnostic potential of the metabolic signature identified. Moreover, complementary -omic approaches, such as exome, transcriptome or methylome of these patients, are needed to further understand the underlying mechanisms in NET development and progression. In particular, the metabolomic profile could be combined with complementary analytical approaches in plasma such as cell-free nucleic acids profiling that might be particularly useful for early diagnostics and patient stratification for personalized clinical management. Plasma -omic profiling has the additional advantage of providing a dynamic characterization of disease biology, which could be eventually utilized, beyond accompanying diagnostics, for targeted prevention or screening, individualized treatment strategies, therapeutic monitoring and prediction of patient’s outcome.

## 4. Material and Methods

### 4.1. Study Population

The study population included patients with advanced, well-differentiated NETs of lung or gastrointestinal origin. Main clinical and pathological features of the study population are summarized in Table S1. Blood samples were obtained for metabolomic analysis from 77 NET patients and 68 non-cancer individuals as the control group. The distribution of gender, age and body mass index (BMI) was similar in the NET and non-cancer cohorts (Table S11). Peripheral blood was collected in sodium EDTA tubes according to standard procedures and fractionated at 3000 rpm for 5 min. Plasma layer was recovered in sterile cryotubes, frozen and stored until use at −80 °C. The study protocol was approved by the institutional ethics committee and all patients provided informed consent prior to study entry.

### 4.2. Multiplatform Metabolic Fingerprinting

A multiplatform non-targeted metabolomics approach was performed to provide a wide coverage of the metabolome under study. Samples were analyzed according to standard protocols through different separation techniques coupled to mass spectrometry: capillary electrophoresis 7100 coupled to a MS with time-of-flight analyzer, TOF-MS 6224 (Agilent Technologies, Santa Clara, CA, USA) (CE−MS), HPLC system 1290 Infinity II coupled with 6545 QTOF MS detector (Agilent Technologies, Santa Clara, CA, USA) (LC−MS) and GC system 7890A coupled to a mass spectrometer 5975C (Agilent Technologies, Santa Clara, CA, USA) (GC−MS) [46,47,48] (see File S1).

#### 4.2.1. Data Processing

The raw data obtained by CE−MS were processed with MassHunter Profinder software version B.08.00, applying the Molecular Feature Extraction (MFE) and Find by Ion (FbI) function by Recursive Feature Extraction (RFE). For LC−MS, the raw data were reprocessed by the MFE with MassHunter Qualitative (B.06.00, Agilent Software, Santa Clara, USA) and DA Reprocessor Offline Utilities B.05.00 (Agilent) and Mass Profiler Professional software (B.14.9 Agilent Software, Santa Clara, USA) to find coeluting adducts and aligned and filtered the data. Raw data files from GC−MS analysis were converted to the appropriate format for quantitative analysis through MassHunter Workstation GC−MS Translator (B.04.01) and deconvolution was carried out through Agilent MassHunter Unknowns Analysis Tool 7.0. For more specific details, see File S1.

#### 4.2.2. Statistical Analysis

After correction (see File S1), the data underwent a Quality Assurance procedure, data normality for every platform was assessed by Kolmogorov−Smirnov and Shapiro−Wilk tests and Levene’s test was used to test for variance ratio. To determine the statistical significance of each metabolite separately, differences between non-cancer individuals and NET cases were evaluated by applying Student’s *t*-test (*p* ≤ 0.05) using MATLAB (R2015a, MathWorks, Natick, MA, USA). Benjamini−Hochberg multiple post-correction method was applied to all *p*-values to control the false positive rate at level α = 0.05.

Multivariate analysis (MVA) was performed in SIMCA 15.0 (Sartorius Stedim Biotech) OPLS-DA model built was used to assess the S-plot and, for variable selection, volcano plots of variable importance in projection (VIP) score and *p*(corr) [49]. For more specific details, see Supplementary Material and Methods.

#### 4.2.3. Annotation and Compound Identification

An initial annotation of features from LC−MS and CE−MS based on the m/z of the compounds showing significant differences in class separation was performed by CEU Mass Mediator tool [50] (see File S1).

To confirm the annotation of the compounds, LC−MS/MS analysis was carried out by data independent analysis (DIA) and the identification of each metabolite was achieved by manual MS/MS spectra interpretation. Some CE−MS annotations could also be confirmed through in-source fragmentation obtained at high fragmentor voltage (200 V) [51] (see File S1).

#### 4.2.4. Targeted Analysis

Standards used and the corresponding sources are included in Table S12. Two calibration curves were prepared according to the solubility of the standards. An aqueous mixture containing arginine, 1-methyladenosine, biliverdin and bilirubin was diluted to 6 different concentration levels (ca. 1 ng/mL to 1 µg/mL) and another mixture containing 5-hydroxyindoleacetic acid, 3-hydroxydodecanedioic acid, linoleoylcarnitine, oleoylcarnitine, sphingosine-1-phosphate, 3-hydroxydodecanoic acid, HETE, ursodeoxycholic acid and ursodeoxycholic acid 3-sulfate was diluted inMeOH/EtOH (1:1). Plasma samples were prepared using the same protocol used for untargeted HPLC/MS analysis [47]. Targeted analysis was performed on an Agilent 1290 Infinity UHPLC (Agilent Technologies, Waldbronn, Germany) system coupled with an Agilent 6460 Triple Quadrupole Mass Spectrometer with an electrospray ionization (ESI) source (HPLC QqQ MS/MS). In the final method, chromatographic separation of compounds was achieved with a Zorbax C8 Eclipse Plus column (Agilent Technologies, 2.1 × 150 mm, 1.8 μm) thermostated at 55 °C. For individual analytes, MS-related parameters were tuned by the Agilent MassHunter Optimizer (software version B.07.00, Agilent Software, Santa Clara, USA) using authentic standards for reference. MassHunter Optimizer automatically optimized the data acquisition parameters for MRM (multiple-reaction monitoring) mode. System control and initial chromatogram review were performed with Agilent MassHunter Qualitative software (version B.08.00, Agilent Software, Santa Clara, USA). Data reprocessing were carried out using Agilent MassHunter QQQ Quantitative software program (version B.09.00, Agilent Software, Santa Clara, USA). Metabolites were quantified according to the response factor of the respective calibration curve.

### 4.3. Metabolite Classification and Pathway Analysis

The discriminant metabolites summarized in Table 1 were classified by biochemical classes and by their relationship with specific molecular pathways (apoptosis, cell cycle, angiogenesis, mTOR pathway, Warburg effect, oxidative stress, tryptophan metabolism, collagen metabolism, carnitine metabolism, methionine cycle, arachidonic acid metabolism, urea cycle, polyamines, and heme metabolism). In order to refine the identification of aberrant molecular pathways in NET patients we analysed our data by Metabolite Pathway Analysis (MPA) and Metabolite Set Enrichment Analysis (MSEA) using MetaboAnalyst 4.0 platform (http://www.metaboanalyst.ca/, accessed on 8 December 2020) [52]. The databases of reference employed were KEGG homo sapiens (Oct 2019) and SMPD [53,54].

### 4.4. Clinical and Molecular Data Analysis

In order to evaluate the diagnostic potential of metabolites, Receiver Operating Characteristic (ROC) curves and Area Under the Curve (AUCs) were assessed, and sensitivity and specificity values were calculated (according to Youden Index) [55]. Associations with relevant clinical features (age, gender, BMI, glycemia and creatinine plasma levels) and common concomitant medications selected by their putative influence in metabolomics (Table S13) were assessed in metabolites with AUC > 0.85, using Fisher’s exact test, chi-squared test or Pearson correlation, as appropriate (*p* < 0.05 were considered significant). Next, logistic regression models were built for each metabolite adjusting for age, gender, glycemia, creatinine plasma levels and significant patient medication selected from association analysis. Metabolites with AUC > 0.85 were considered as potential biomarkers. Additionally, differential metabolites from OPLS-DA models with VIP >1.0 and |*p*(corr)| > 0.5 were also considered as potential biomarkers.

### 4.5. Heatmap and Hierarchical Clustering

Heatmaps were conducted with the log10 value of each metabolite levels in plasma samples. Unsupervised hierarchical clustering was performed for metabolites and patients using Pearson correlation and average as linkage method. Both were conducted using the Morpheus Software (Broad Institute; https://software.broadinstitute.org/morpheus, accessed on 18 December 2020).

## 5. Conclusions

In conclusion, untargeted plasma metabolomic profiling of NET patients, that integrated metabolic data acquired by GC, CE and LC in both polarity modes coupled to MS, has identified a distinct metabolic signature of potential clinical use. Indeed, our study has identified and validated a reduced set of metabolites of high diagnostic accuracy that may improve the specific detection of NETs. Differential metabolites were related with classical cancer pathways (apoptosis, cell cycle) and NET signaling (tryptophan metabolism, angiogenesis, mTOR). In addition, MPA/MSEA analysis of these metabolites has revealed new enriched metabolic pathways in NETs, related with the TCA cycle and with arginine, pyruvate or glutathione metabolism, which have distinct implications in oncogenesis and may open innovative avenues of clinical research, including the identification of potential novel targets for therapy. This is to our knowledge the most comprehensive metabolic profiling study performed to date in NETs and provides very valuable information to develop useful biomarkers for the management of these patients in clinical practice.

## Figures and Tables

**Table 1:** List of the statistically significant annotated metabolites discriminating between the plasma profiles of control (*n* = 68) and NET patients (*n* = 77) with their statistical characteristics after UVDA and MVDA (percentage of change, *p*-value, *p*(corr) and VIP) and analytical descriptors (measured mass and its deviation from the theoretical one, experimental retention time, analytical platform on which it has been detected, identification source where DB corresponds to database result, confidence level for identification according to the metabolomics standards initiative and its corresponding Human Metabolome Database (HMDB) code (http://www.hmdb.ca/, accessed on 16 December 2020)). (* = Multiple identification options).

Compound	Formula	Mass (Da)	RT (Min)	% Change	*p-*Value	*p*(corr)	VIP	Mass Error (ppm)	Analytical Platform	Identification Source	Confidence Level	HMDB Code
**Amines**												
Triethylamine	C_6_H_15_N	101.1204	10.28	−53	0.0009			0	CE-MS	DB	3	HMDB0032539
**Amino acids, peptides and analogues**												
Arginine	C_6_H_14_N_4_O_2_	174.1131	9.98	243	4.58 × 10^−35^	−0.80	2.33	15	CE-MS LC-MS(+)	DB	2	HMDB0000517
Arg-Val	C_11_H_23_N_5_O_3_	273.1822	9.95	66	0.0004			8	CE-MS	DB	3	HMDB0028722
Aspartate	C_4_H_7_NO_4_	133.0375	13.80	−32	4.98 × 10^−13^			2	CE-MS	DB	2	HMDB0000191
Cys-Gly	C_5_H_10_N_2_O_3_S	178.0412	12.19	−29	8.00 × 10^−9^			0	CE-MS	DB	3	HMDB0000078
Cys-Gly disulfide	C_8_H_15_N_3_O_5_S_2_	297.0455	12.19	−41	1.39 × 10^−8^	0.49	1.63	1	CE-MS	DB	3	HMDB0000709
Cysteineglutathione disulfide	C_13_H_22_N_4_O_8_S_2_	426.0912	13.95	−37	7.40 × 10^−6^			8	CE-MS	DB	3	HMDB0000656
Dimethyl-Arginine (symmetric)	C_8_H_18_N_4_O_2_	202.1428	10.67	25	0.0006			1	CE-MS	DB	2	HMDB0003334
GalactosylhydroxyLys	C_12_H_24_N_2_O_8_	324.1558	11.49	42	0.0014			8	CE-MS	DB	3	HMDB0000600
gamma-Glu-orn *	C_10_H_19_N_3_O_5_	261.1335	11.30	34	0.0103			4	CE-MS	DB	3	HMDB0002248
Glu-Ala *	C_8_H_14_N_2_O_5_	218.0904	14.40	135	4.38 × 10^−14^			1	CE-MS	DB	3	HMDB0006248
Glu-Arg	C_11_H_21_N_5_O_5_	303.1554	11.49	87	6.28 × 10^−14^	−0.52	1.55	4	CE-MS	DB	3	HMDB0028813
Glu-Asp	C_9_H_14_N_2_O_7_	262.0801	3.78	54	0.0036			7	LC-MS(−)	MSMS	2	HMDB0030419
Glu-hyp	C_10_H_16_N_2_O_6_	260.0993	12.98	37	5.12 × 10^−7^			6	CE-MS	DB	3	HMDB0011161
Glu-Lys/Ɛ-Glu-Lys	C_11_H_21_N_3_O_5_	275.1481	12.32	67	1.20 × 10^−9^			2	CE-MS	DB	3	HMDB0029154
Glu-Lys/Ɛ-Glu-Lys	C_11_H_21_N_3_O_5_	275.1481	11.37	58	1.55 × 10^−7^			2	CE-MS	DB	3	HMDB0029155
Glutamine	C_5_H_10_N_2_O_3_	146.0691	1.34	69	0.0006			1	LC-MS(+) LC-MS(−)	MSMS	2	HMDB0000641
Glu-Val	C_10_H_18_N_2_O_5_	246.1217	14.68	48	0.0009			1	CE-MS LC-MS(+)	DB	3	HMDB0028832
Glycine	C_2_H_5_NO_2_	75.0320	9.75	32	0.0202			-	GC-MS	Fiehn	2	HMDB0000123
Gly-Pro	C_7_H_12_N_2_O_3_	172.0831	15.89	−20	0.0275			10	CE-MS	DB	3	HMDB0000721
Homocitrulline	C_7_H_15_N_3_O_3_	189.1118	13.52	44	0.0027			3	CE-MS	DB	3	HMDB0000679
Iminodiacetic acid	C_4_H_7_NO_4_	133.0375	12.87	−37	0.0158			-	GC-MS CE-MS	Fiehn	2	HMDB0011753
Indoleacetyl glutamine	C_15_H_17_N_3_O_4_	303.1219	1.70	203	1.43 × 10^−6^	−0.58	2.05	2	LC-MS(−) LC-MS(+)	DB	3	HMDB0013240
Leu-hyp	C_11_H_20_N_2_O_4_	244.1423	2.00	87	0.0018			2	LC-MS(−)	DB	3	HMDB0028867
Leu-Phe	C_15_H_22_N_2_O_3_	278.1630	2.14	Presented in cancer group	3.54 × 10^−5^	−0.60	2.45	0	LC-MS(−)	DB	3	HMDB0013243
Lys-Asp *	C_10_H_19_N_3_O_5_	261.1335	11.30	34	0.0103			4	CE-MS	DB	3	HMDB0028947
Methionine S-oxide	C_5_H_11_NO_3_S	165.0478	14.06	110	3.30 × 10^−9^			11	CE-MS	DB	2	HMDB0002005
N2-Methyl-lysine	C_7_H_16_N_2_O_2_	160.1208	10.67	−77	6.97 × 10^−9^			2	CE-MS	DB	2	HMDB0002038
N2-Methylproline	C_6_H_11_NO_2_	129.0791	14.59	−43	0.0031			1	CE-MS	DB	2	HMDB0059649
N6-Acetyl-hydroxy-lysine *	C_8_H_16_N_2_O_4_	204.1103	12.78	−55	0.0002			3	CE-MS	DB	3	HMDB0033891
N-acetyl-lysine	C_8_H_16_N_2_O_3_	188.1155	13.70	20	0.0002			3	CE-MS	DB	2	HMDB0000206
Ornithine	C_5_H_12_N_2_O_2_	132.0906	9.66	−39	5.58 × 10^−19^	0.65	1.65	6	CE-MS	DB	2	HMDB0000214
Phenylalanine	C_9_H_11_NO_2_	165.0770	13.88	−58	0.0050			12	CE-MS GC-MS	DB	2	HMDB0000159
Pipecolic acid	C_6_H_11_NO_2_	129.0790	10.14	49	0.0307			-	GC-MS	Fiehn	2	HMDB0000716
Pyroglutamine	C_5_H_8_N_2_O_2_	128.0583	11.48	68	1.41 × 10^−5^			2	CE-MS	DB	3	HMDB0062558
Ser-Ala *	C_6_H_12_N_2_O_4_	176.0789	13.07	−38	1.37 × 10^−8^			4	CE-MS	DB	3	HMDB0029032
Ser-hyp *	C_8_H_14_N_2_O_5_	218.0904	14.40	135	4.38 × 10^−14^			1	CE-MS	DB	3	HMDB0029040
Ser-Val *	C_8_H_16_N_2_O_4_	204.1103	12.78	−55	0.0002			3	CE-MS	DB	3	HMDB0029052
Stearoyl-tyrosine *	C_27_H_45_NO_4_	447.3349	5.15	−66	9.67 × 10^−17^	0.75	3.74	1	LC-MS(+)	DB	3	HMDB0062343
Suberylglycine	C_10_H_17_NO_5_	231.1106	1.19	Presented in cancer group	0.0001	−0.73	3.82	1	LC-MS(−)	DB	3	HMDB0000953
Thr-Ala	C_7_H_14_N_2_O_4_	190.0954	13.83	90	5.09 × 10^−11^			0	CE-MS	DB	3	HMDB0029054
Thr-Gly *	C_6_H_12_N_2_O_4_	176.0789	13.07	−38	1.37 × 10^−8^			4	CE-MS	DB	3	HMDB0029061
Trp-Phe	C_20_H_21_N_3_O_3_	351.1582	2.32	74	3.73 × 10^−11^	−0.63	1.56	1	LC-MS(−)	DB	3	HMDB0029090
Val-Leu	C_11_H_22_N_2_O_3_	230.1616	12.83	51	0.0012			6	CE-MS	DB	3	HMDB0029131
**Benzene and substituted derivatives**												
Mandelic acid	C_8_H_8_O_3_	152.0473	2.28	335	0.0037			3	LC-MS(−)	DB	3	HMDB0000703
3-phenylprop-2-en-1-yloxysulfonic acid	C_9_H_10_O_4_S	214.0299	2.77	118	0.0005			2	LC-MS(−)	DB	3	HMDB0135284
**Carbohydrates and carbohydrate conjugates**												
Allose	C_6_H_12_O_6_	180.0633	17.10	139	1.32 × 10^−14^	0.62	1.44	-	GC-MS	Fiehn	2	HMDB0001151
Glucose	C_6_H_12_O_6_	180.0633	17.45	174	1.98 × 10^−15^	0.62	1.64	-	GC-MS	Fiehn	2	HMDB0000122
Glycerol	C_3_H_8_O_3_	92.0473	9.28	45	0.0000			-	GC-MS	Fiehn	2	HMDB0000131
Mannitol	C_6_H_14_O_6_	182.0790	17.59	196	0.0017			-	GC-MS	Fiehn	2	HMDB0000765
Phenylglucuronide	C_12_H_14_O_7_	270.0739	0.93	308	0.0052	−0.52	2.37	2	LC-MS(−)	DB	3	HMDB0060014
PYRANOSE (glucose/altrose/galactose/talose)	C_6_H_12_O_6_	180.0633	17.24	194	1.61 × 10^−16^	0.63	1.71	-	GC-MS	Fiehn	2	
**Carboximidic acids and derivatives**												
Acetylspermidine	C_9_H_21_N_3_O	187.1671	9.15	38	6.27 × 10^−6^			7	CE-MS	DB	3	HMDB0001276
**Carboxylic acids and derivatives**												
1-Aminocyclohexanecarboxylic acid	C_7_H_13_NO_2_	143.0944	13.89	−32	0.0366			1	CE-MS	DB	3	HMDB0038249
di-Hydroxymelatonin *	C_13_H_16_N_2_O_4_	264.1110	1.34	46	0.0268			2	LC-MS(−)	DB	3	HMDB0061136
Edetic Acid	C_10_H_16_N_2_O_8_	292.0906	0.24	35	0.0001			1	LC-MS(+)	DB	3	HMDB0015109
Isocitric acid Citric acid	C_6_H_8_O_7_	192.0270	0.23	64	5.83 × 10^−5^			0	LC-MS(−)	MSMS	2	HMDB0000193HMDB0000094
**Diazines/Pyrimidines and pyrimidine derivatives**												
5,6-Dihydrothymine	C_5_H_8_N_2_O_2_	128.0570	12.22	56	0.0169			12	CE-MS	DB	3	HMDB0000079
**Fatty acyls**												
3-carboxy-4-methyl-5-propyl-2-furanpropanoic acid (CMPF)	C_12_H_16_O_5_	240.0998	3.78	69	0.004			2	LC-MS(+)	MSMS	2	HMDB0061112
8-amino-7-oxo-nonanoic acid *	C_9_H_17_NO_3_	187.1208	2.06	418	0.0220			2	LC-MS(−)	DB	3	
Arachidonic acid	C_20_H_32_O_2_	304.2402	7.13	62	1.59 × 10^−5^			1	LC-MS(−)	MSMS	2	HMDB0001043
beta-Phenylalanoyl- CoA *	C_30_H_45_N_8_O_17_P_3_S	914.1836	2.83	73	7.07 × 10^−3^			1	LC-MS(−)	DB	3	
beta-Phenylalanoyl-CoA *	C_30_H_45_N_8_O_17_P_3_S	914.1836	3.62	65	8.78 × 10^−3^			0	LC-MS(−)	DB	3	
DG(31:0)	C_34_H_66_O_5_	554.4910	8.18	−37	0.0008			0	LC-MS(+)	DB	3	HMDB0093505
Docosapentaenoic acid	C_22_H_34_O_2_	330.2558	7.25	75	6.87 × 10^−9^	−0.55	1.51	1	LC-MS(−)	DB	3	HMDB0006528
Dodecenedioic acid	C_12_H_20_O_4_	228.1362	3.50	191	9.87× 10^−8^	−0.60	2.24	2	LC-MS(−)	DB	3	HMDB0000933
Eicosapentaenoic acid	C_20_H_30_O_2_	302.2246	6.77	65	3.88 × 10^−4^			2	LC-MS(−)	DB	3	HMDB0001999
Eicosatrienoic acid	C_20_H_34_O_2_	306.2558	7.39	82	1.85 × 10^−10^			1	LC-MS(−)	DB	3	HMDB0010378
Eicosenoic acid	C_20_H_38_O_2_	310.2871	8.34	79	2.75 × 10^−10^	−0.51	1.50	1	LC-MS(−)	DB	3	HMDB0002231
Glucosylgalactosylhydroxylysine	C_18_H_34_N_2_O_13_	486.2093	12.38	46	1.73 × 10^−5^			7	CE-MS	DB	3	HMDB0000585
HETE	C_20_H_32_O_3_	320.2351	5.92	Presented in cancer group	1.24 × 10^−5^	−0.63	2.64	0	LC-MS(−)	MSMS	2	HMDB0060101
MG(18:2)	C_21_H_38_O_4_	354.2770	6.81	116	0.0007			2	LC-MS(+)	MSMS	2	HMDB0011538
MG(20:0)	C_23_H_46_O_4_	386.3396	7.79	−86	1.30 × 10^−12^			5	LC-MS(+)	DB	3	HMDB0072859
N-palmitoyl glutamic acid *	C_21_H_39_NO_5_	385.2828	4.13	62	0.004			0	LC-MS(+)	DB	3	
Oleic acid	C_18_H_34_O_2_	282.2559	20.47	67	0.0013			-	GC-MS LC-MS(+)	Fiehn	2	HMDB0000207
Vaccenic acid	C_18_H_34_O_2_	282.2559	20.56	23	0.0158			-	GC-MS	Fiehn	2	HMDB0041480
3-hydroxy-5-octenoylcarnitine	C_15_H_27_NO_5_	301.1889	4.23	Presented in cancer group	3.57 × 10^−2^	−0.72	3.96	3	LC-MS(−)	DB	3	
3-Hydroxy-5-tetradecenoylcarnitine *	C_21_H_39_NO_5_	385.2828	4.13	62	0.004			0	LC-MS(+)	DB	3	HMDB0013330
9-Decenoylcarnitine	C_17_H_31_NO_4_	313.2232	12.99	−15	0.0325			7	CE-MS	DB	3	HMDB0013205
Arachidonoylcarnitine *	C_27_H_45_NO_4_	447.3349	5.15	−66	9.67 × 10^−17^	0.75	3.74	1	LC-MS(+)	DB	3	HMDB0062343
α-Linolenyl carnitine	C_25_H_43_NO_4_	421.3192	4.91	−41	1.79 × 10^−8^	0.53	2.26	0	LC-MS(+)	DB	3	HMDB0006319
Linoleyl carnitine	C_25_H_45_NO_4_	423.3349	5.14	−59	4.03 × 10^−16^	0.73	3.40	0	LC-MS(+)	DB	3	HMDB0006469
Oleoylcarnitine Elaidic carnitine	C_25_H_47_NO_4_	425.3504	5.43	−41	1.41 × 10^−8^	0.51	2.40	1	LC-MS(+)	MSMS	2	HMDB0006351 HMDB0006464
**Flavonoids**												
Anthraniloyl-CoA	C_28_H_41_N_8_O_17_P_3_S	886.1523	0.23	186	3.47 × 10^−21^	−0.69	2.39	3	LC-MS(−)	DB	3	
**Glycerolipids**												
11-Oxo-androsterone glucuronide	C_25_H_36_O_9_	480.2359	3.43	−45	8.06 × 10^−6^	0.53	2.06	1	LC-MS(−)	DB	3	HMDB0010338
**Glycerophospholipids**												
LPC(16:0)-OH	C_24_H_50_NO_8_P	511.3274	4.12	25	0.008			0	LC-MS(+) LC-MS(−)	MSMS	2	
LPC(16:0)-OH	C_24_H_50_NO_8_P	511.3274	4.21	37	0.0005			0	LC-MS(+) LC-MS(−)	MSMS	2	
LPC(18:0)-OH	C_26_H_54_NO_8_P	539.3587	4.71	27	0.0100			0	LC-MS(+) LC-MS(−)	MSMS	2	
LPC(18:2)-OH	C_26_H_50_NO_8_P	535.3274	4.41	Presented in cancer group	2.19 × 10^−6^			0	LC-MS(+) LC-MS(−)	MSMS	2	
LPA(13:0)	C_16_H_33_O_7_P	368.1963	5.23	−57	4.95 × 10^−3^	0.56	2.29	7	LC-MS(−)	DB	3	HMDB0114760
LPC(22:1)	C_30_H_60_NO_7_P	577.4107	6.99	66	2.14 × 10^−5^			2	LC-MS(−)	MSMS	2	HMDB0010399
LPE(16:0)	C_21_H_44_NO_7_P	453.2855	5.65	25	0.008			0	LC-MS(+)	MSMS	2	HMDB0011473
LPE(20:5)	C_25_H_42_NO_7_P	499.2699	5.08	179	2.80 × 10^−6^			0	LC-MS(+)	MSMS	2	HMDB0011489
LPE(20:5)	C_25_H_42_NO_7_P	499.2699	5.16	55	0.001			0	LC-MS(+) LC-MS(−)	MSMS	2	HMDB0011489
LPE(22:6)	C_27_H_44_NO_7_P	525.2855	5.37	28	0.001			0	LC-MS(+)	MSMS	2	HMDB0011526
LPE(22:6)	C_27_H_44_NO_7_P	525.2855	5.45	39	0.0005			0	LC-MS(+)	MSMS	2	HMDB0011496
LPE(P-16:0)	C_21_H_44_NO_6_P	437.2906	5.83	36	0.002			0	LC-MS(+)	DB	3	HMDB0011152
LPI(16:1)	C_25_H_47_O_12_P	570.2805	5.51	97	2.63 × 10^−3^			1	LC-MS(−)	MSMS	2	
LPS(18:0)	C_24_H_48_NO_9_P	525.3066	6.71	104	9.62 × 10^−6^			2	LC-MS(−)	MSMS	2	
PC(32:0)	C_40_H_80_NO_8_P	733.5622	10.47	51	3.91 × 10^−7^			2	LC-MS(+)	DB	3	HMDB0007871
PC(38:2)	C_46_H_88_NO_8_P	813.6247	11.94	39	0.0004			0	LC-MS(+)	MSMS	2	HMDB0007987
PC(38:5)	C_46_H_82_NO_8_P	807.5778	9.82	−28	5.05 × 10^−5^			2	LC-MS(+)	MSMS	2	HMDB0008156
PE(34:2) PE(O-34:3)	C_39_H_74_NO_7_P	699.5203	10.25	−33	0.001			1	LC-MS(+)	MSMS	2	HMDB0011343
PE(38:6)	C_43_H_74_NO_8_P	763.5151	9.31	−31	0.0087			1	LC-MS(+)	MSMS	2	HMDB0009294
PG(20:2)	C_26_H_49_O_9_P	536.3114	7.41	−58	3.44 × 10^−11^	0.59	2.33	4	LC-MS(−)	DB	3	
PG(28:0)	C_34_H_67_O_10_P	666.4471	7.64	115	8.69 × 10^−15^	−0.61	2.75	5	LC-MS(+)	DB	3	HMDB0116681
PS(39:5)	C_45_H_78_NO_10_P	823.5363	10.46	25	1.60 × 10^−7^			5	LC-MS(+)	DB	3	
**Hydroxy acids and derivatives**												
3-Hydroxydodecanedioic acid	C_12_H_22_O_5_	246.1467	2.79	Presented in cancer group	2.42 × 10^−6^	−0.68	2.97	0	LC-MS(−)	MSMS	2	HMDB0000413
3-Hydroxydodecanoic acid	C_12_H_24_O_3_	216.1725	4.62	Presented in cancer group	2.32 × 10^−11^	−0.72	2.79	2	LC-MS(−)	MSMS	2	HMDB0000387
**Imidazoles**												
Methylimidazole	C_4_H_6_N_2_	82.0538	11.48	64	6.41 × 10^−6^			9	CE-MS	DB	3	
Urocanate Nicotinamide N-oxide	C_6_H_6_N_2_O_2_	138.0435	11.02	24	0.001			4	CE-MS	DB	3	HMDB0034174 HMDB0002730
**Imidazopyrimidines/Purines and purine derivatives**												
Hypoxanthine	C_5_H_4_N_4_O	136.0402	14.19	−34	8.20 × 10^−6^			13	CE-MS	DB	2	HMDB0000157
Indoles and derivatives												
3-Indoleacetic acid	C_10_H_9_NO_2_	175.0633	17.94	131	2.62 × 10^−7^			-	GC-MS	Fiehn	2	HMDB0000197
5-Hydroxyindole	C_8_H_7_NO	133.0527	0.76	37	3.82 × 10^−2^			1	LC-MS(−)	DB	3	HMDB0059805
5-Hydroxyindoleacetaldehyde	C_10_H_9_NO_2_	175.0633	2.51	150	2.63 × 10^−5^	−0.51	2.55	1	LC-MS(+)	MSMS	2	HMDB0004073
5-Hydroxyindoleacetic acid	C_10_H_9_NO_3_	191.0582	0.80	Presented in cancer group	3.28 × 10^−8^			0	LC-MS(+)	MSMS	2	HMDB0000763
**Lactones**												
N-(4-Coumaroyl)-homoserine lactone	C_13_H_13_NO_4_	247.0845	1.34	55	0.0006			2	LC-MS(+)	DB	3	
**Organic acids and derivatives**												
(Homo)2-aconitate*	C_8_H_10_O_6_	202.0477	0.26	−75	7.87 × 10^−59^	0.84	2.98	11	LC-MS(−)	DB	3	
Lactic acid	C_3_H_6_O_3_	90.0317	6.06	−77	4.72 × 10^−36^	−0.92	2.58	-	GC-MS	Fiehn	2	HMDB0001311
Pyruvic acid	C_3_H_4_O_3_	88.0160	5.89	−58	1.06 × 10^−14^	−0.69	1.78	-	GC-MS	Fiehn	2	HMDB0000243
Succinylacetoacetate *	C_8_H_10_O_6_	202.0477	0.26	−75	7.87 × 10^−59^	0.84	2.98	11	LC-MS(−)	DB	3	HMDB0240258
**Organic sulfuric acids and derivatives**												
Indoxylsulfuric acid	C_8_H_7_NO_4_S	213.0095	1.00	42	3.47 × 10^−2^			1	LC-MS(−)	MSMS	2	HMDB0000682
p-Phenolsulfonic acid	C_6_H_6_O_4_S	173.9986	0.65	174	3.45 × 10^−3^			1	LC-MS(−)	MSMS	2	HMDB0060015
**Organonitrogen compounds**												
Phosphocholine	C_5_H_14_NO_4_P	183.0660	5.37	32	0.003			1	LC-MS(+)	MSMS	2	
**Organooxygen compounds**												
4-Hydroxycyclohexylcarboxylic acid	C_7_H_12_O_3_	144.0786	0.76	789	5.08 × 10^−10^	−0.71	2.99	1	LC-MS(−)	DB	3	HMDB0001988
Acetyl-N-formyl-5-methoxykynurenamine (AFMK) *	C_13_H_16_N_2_O_4_	264.1110	1.34	46	0.0268			2	LC-MS(−)	DB	3	HMDB0004259
**Phenols**												
4-Methylcatechol	C_7_H_8_O_2_	124.0524	1.23	−43	1.68 × 10^−2^			2	LC-MS(−)	DB	3	HMDB0000873
**Prenol lipids**												
Retinol	C_20_H_30_O	286.2296	7.04	34	0.0003			2	LC-MS(+)	MSMS	2	HMDB0000305
**Purine nucleosides**												
1-Methyladenosine	C_11_H_15_N_5_O_4_	281.1132	12.49	41	3.68 × 10^−16^	−0.58	1.20	3	CE-MS	DB	2	HMDB0003331
**Pyridines and derivatives**												
Norcotinine	C_9_H_10_N_2_O	162.0790	11.48	34	0.0039			2	CE-MS	DB	3	HMDB0001297
Piperideine	C_5_H_9_N	83.0739	13.77	28	0.0239			5	CE-MS	DB	3	
Serotonine	C_10_H_12_N_2_O	176.0954	11.40	228	0.0007			3	CE-MS	DB	2	HMDB0001046
**Quinolines and derivatives**												
8-Hydroxycarteolol	C_16_H_24_N_2_O_4_	308.1732	13.90	−31	0.0246			1	CE-MS	DB	3	HMDB0060990
Quinoline	C_9_H_7_N	129.0578	2.51	99	0.0008			1	LC-MS(+)	DB	3	HMDB0033731
**Sphingolipids**												
Cer(35:0)	C_35_H_69_NO_3_	551.5277	11.39	58	4.24 × 10^−8^			3	LC-MS(−)	DB	3	
Cer(36:1)	C_36_H_71_NO_3_	565.5434	11.74	42	0.0003			1	LC-MS(+)	DB	3	HMDB0004950
SM(36:0)	C_41_H_85_N_2_O_6_P	732.6145	10.14	41	0.0014			1	LC-MS(+)	MSMS	2	HMDB0012087
Sphingosine-1-phosphate	C_18_H_38_NO_5_P	379.2488	5.02	−30	2.17 × 10^−8^	0.50	2.06	0	LC-MS(+)	MSMS	2	HMDB0000277
**Steroids and steroid derivatives**												
12a-Hydroxy-3-oxocholadienic acid	C_24_H_34_O_4_	386.2457	4.27	144	3.66 × 10^−6^	−0.54	2.12	0	LC-MS(−)	MSMS	2	HMDB0000385
Biliverdin	C_33_H_34_N_4_O_6_	582.2478	4.73	300	1.47 × 10^−16^	−0.72	2.62	0	LC-MS(−)	DB	3	HMDB0001008
Hydroxy-3-oxo-4-cholestenoate	C_27_H_42_O_4_	430.3083	5.53	54	2.78 × 10^−6^				LC-MS(+) LC-MS(−)	MSMS	2	
Calcitroic acid	C_23_H_34_O_4_	374.2457	7.39	84	6.25 × 10^−11^	−0.54	1.76	6	LC-MS(−)	DB	3	HMDB0006472
Chenodeoxycholic acid 3-glucuronide *	C_30_H_48_O_10_	568.3248	4.33	94	2.10 × 10^−3^			1	LC-MS(−)	DB	3	
Cholestane-3,7,12,24,25-pentol	C_27_H_48_O_5_	452.3501	7.77	−56	3.81 × 10^−10^	0.56	2.03	2	LC-MS(−)	DB	3	HMDB0000483
Cholestane-3,7,12,25-tetrol-3-glucuronide	C_33_H_56_O_10_	612.3873	4.71	97	4.89 × 10^−5^			0	LC-MS(−)	DB	3	HMDB0010355
Cortisone acetate	C_23_H_30_O_6_	402.2042	3.86	63	5.89 × 10^−4^			9	LC-MS(−)	DB	3	HMDB0015459
Dehydroepiandrosterone 3-glucuronide Dehydroisoandrosterone 3-glucuronide	C_25_H_36_O_8_	464.2410	3.91	−51	1.01 × 10^−5^			2	LC-MS(−)	DB	3	HMDB0010348 HMDB0010327
Deoxycholic acid 3-glucuronide *	C_30_H_48_O_10_	568.3248	4.33	94	2.10 × 10^−3^			1	LC-MS(−)	DB	3	HMDB0002596
ecdysone 25-O-D-glucopyranoside	C_33_H_54_O_11_	626.3666	4.41	88	1.02 × 10^−4^			1	LC-MS(−)	DB	3	
Pregnanediol	C_21_H_36_O_2_	320.2715	6.35	−51	4.21 × 10^−5^			0	LC-MS(−)	DB	3	HMDB0004025
Pregnanolone sulfate	C_21_H_34_O_5_S	398.2127	3.64	55	5.27 × 10^−3^			0	LC-MS(−)	DB	3	HMDB0240591
Ursodeoxycholic acid	C_24_H_40_O_4_	392.2927	4.34	188	3.93 × 10^−2^	−0.51	2.35	1	LC-MS(−)	MSMS	2	HMDB0000946
Ursodeoxycholic acid 3-sulfate	C_24_H_40_O_7_S	472.2494	3.75	130	4.38 × 10^−3^	−0.54	2.36	1	LC-MS(−)	DB	3	HMDB0002642
**Sterol lipids**												
24-Hydroxygeminivitamin D3	C_32_H_54_O_5_	518.3971	6.79	−37	2.82 × 10^−7^			1	LC-MS(+)	DB	3	
**Tetrapyrroles and derivatives**												
Bilirubin	C_33_H_36_N_4_O_6_	584.2635	3.85	674	3.79 × 10^−8^	−0.68	2.63	4	LC-MS(−) LC-MS(+)	MSMS	2	HMDB0000054

## Data Availability

The data presented in this study are available from the corresponding author upon reasonable request.

## Supplementary Materials

The following are available online at https://www.mdpi.com/article/10.3390/cancers13112634/s1, Figure S1: PCA-X score plots, Figure S2: PCA-X unsupervised models, Figure S3: NETs show a specific metabolomic profile, Figure S4: Pathway analysis of 48 metabolites with diagnostic potential, Figure S5: Pathway classification of differential plasma metabolites in NET patients, Table S1: Clinical, biochemical and pathological features of NET population, Table S2: Metabolites with biomarker potential in NET patients, Table S3: Drugs as potential confounding variables, Table S4: Logistic regression analysis of metabolites with biomarker potential in the plasma of NET patients, Table S5: Metabolites candidates for targeted validation, Table S6: Validation of potential diagnostic biomarkers of NETs, Table S7: Relevant metabolic pathways related to the identified differential plasma metabolites in NET patients by Metabolite Pathway Analysis (MPA), Table S8: Relevant metabolic pathways related to the identified differential plasma metabolites in NET patients by Metabolite Set Enrichment Analysis (MSEA), Table S9: Relevant metabolic pathways related to the diagnostic biomarker metabolites in NET patients by Metabolite Pathway Analysis (MPA), Table S10: Relevant metabolic pathways related to the diagnostic biomarker metabolites in NET patients by Metabolite Set Enrichment Analysis (MSEA), Table S11: Distribution of age, gender and body mass index in NET patients and non-cancer individuals, Table S12: Standards and the corresponding sources used for the targeted analysis, Table S13: Drug intake of NET and non-cancer patients, File S1: Supplementary Materials and Methods.
