# Supplementary material for: Comprehensive Plasma Metabolomic Profile of Patients with Advanced Neuroendocrine Tumors (NETs). Diagnostic and Biological Relevance
Source: Cancers (Basel). 2021 May 27;13(11):2634. doi: 10.3390/cancers13112634 (PMC8197817; doi:10.3390/cancers13112634)
Supplement: Supplementary file 1 [file cancers-13-02634-s001.zip › cancers-1222208-4-1supplementary/cancers-1222208-4-supplementary-figures.pptx]

## Slide 1
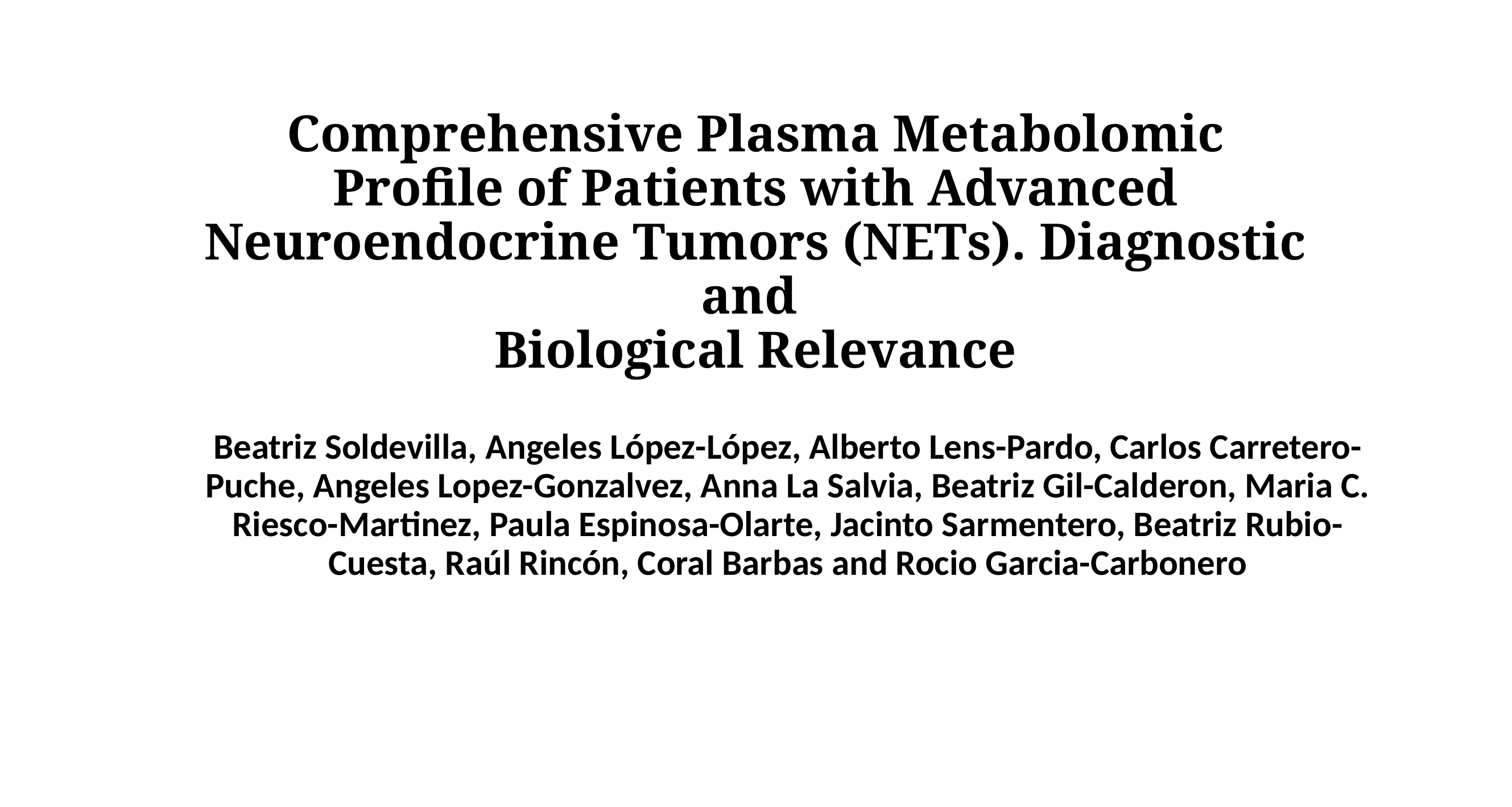

# Comprehensive Plasma Metabolomic Profile of Patients with Advanced Neuroendocrine Tumors (NETs). Diagnostic and Biological Relevance
Beatriz Soldevilla, Angeles López-López, Alberto Lens-Pardo, Carlos Carretero-Puche, Angeles Lopez-Gonzalvez, Anna La Salvia, Beatriz Gil-Calderon, Maria C. Riesco-Martinez, Paula Espinosa-Olarte, Jacinto Sarmentero, Beatriz Rubio-Cuesta, Raúl Rincón, Coral Barbas and Rocio Garcia-Carbonero

## Slide 2
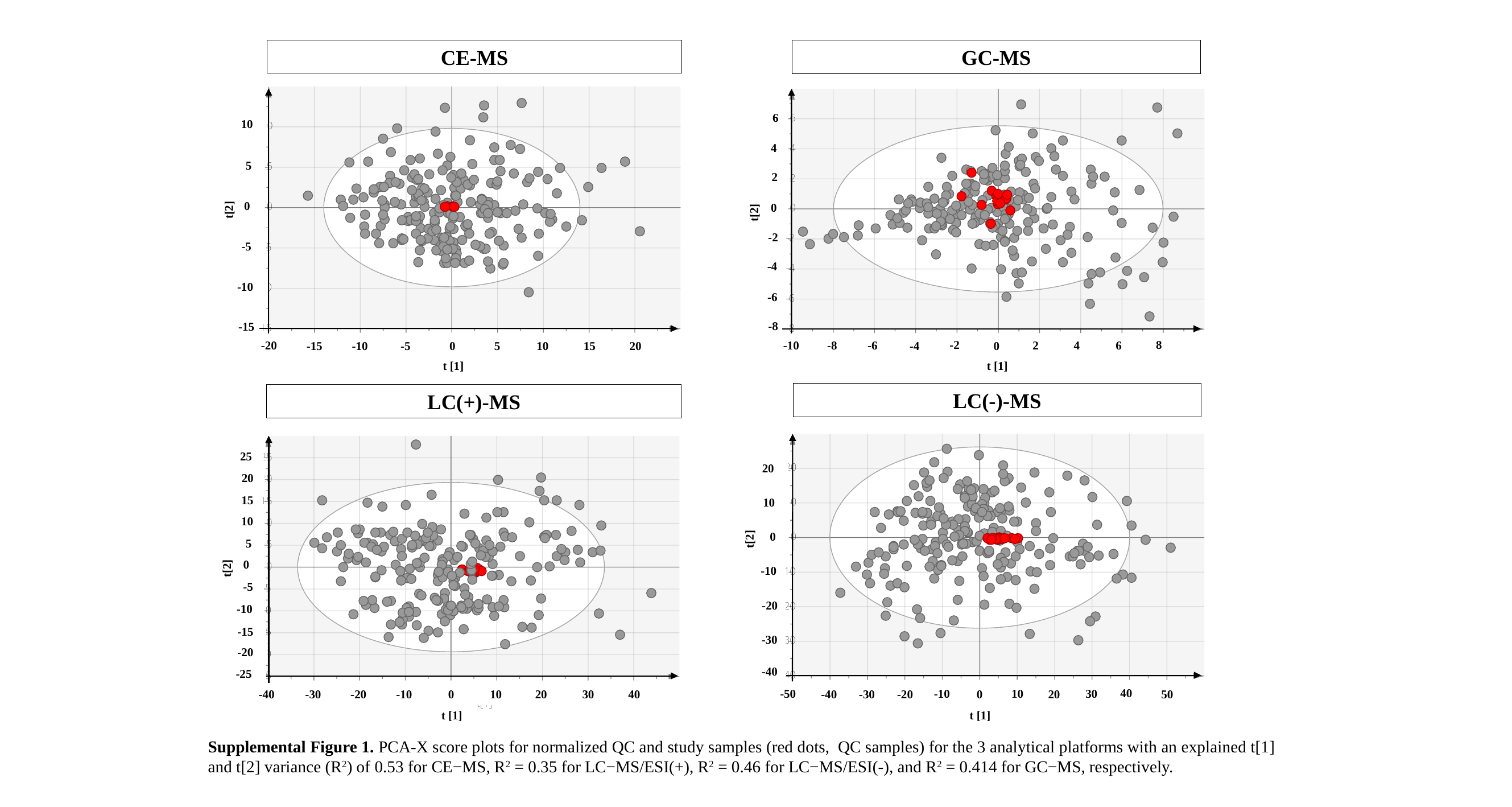

CE-MS
GC-MS
6
10
4
5
2
0
t[2]
0
t[2]
-2
-5
-4
-10
-6
-8
-15
8
-2
-10
-20
2
6
-8
-6
4
5
15
-15
-10
10
20
0
0
-4
-5
t [1]
t [1]
LC(-)-MS
LC(+)-MS
25
20
15
10
5
0
t[2]
-5
-10
-15
-20
-25
10
30
-30
-20
20
40
0
-10
-40
20
10
0
t[2]
-10
-20
-30
-40
40
-10
-50
10
30
-40
-30
20
50
0
-20
t [1]
t [1]
Supplemental Figure 1. PCA-X score plots for normalized QC and study samples (red dots, QC samples) for the 3 analytical platforms with an explained t[1] and t[2] variance (R2) of 0.53 for CE−MS, R2 = 0.35 for LC−MS/ESI(+), R2 = 0.46 for LC−MS/ESI(-), and R2 = 0.414 for GC−MS, respectively.

## Slide 3
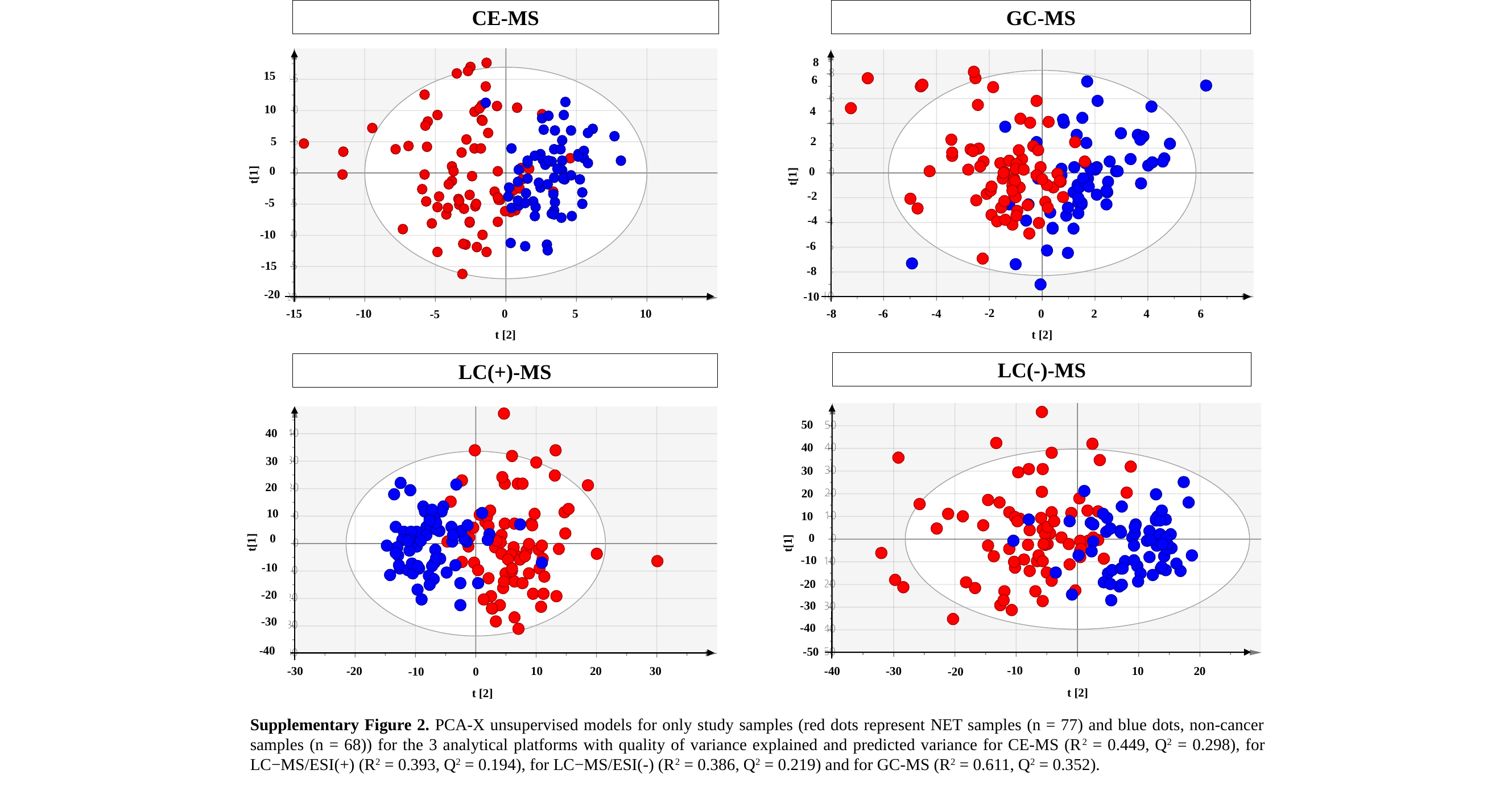

CE-MS
GC-MS
8
15
6
10
4
2
5
0
0
t[1]
t[1]
-2
-5
-4
-10
-6
-15
-8
-20
-10
-2
2
6
-8
-6
4
5
-15
-10
10
0
0
-4
-5
t [2]
t [2]
LC(-)-MS
LC(+)-MS
50
40
40
30
30
20
20
10
10
0
0
t[1]
t[1]
-10
-10
-20
-20
-30
-30
-40
-40
-50
-10
10
-40
-30
20
0
10
30
-30
-20
20
-20
0
-10
t [2]
t [2]
Supplementary Figure 2. PCA-X unsupervised models for only study samples (red dots represent NET samples (n = 77) and blue dots, non-cancer samples (n = 68)) for the 3 analytical platforms with quality of variance explained and predicted variance for CE-MS (R2 = 0.449, Q2 = 0.298), for LC−MS/ESI(+) (R2 = 0.393, Q2 = 0.194), for LC−MS/ESI(-) (R2 = 0.386, Q2 = 0.219) and for GC-MS (R2 = 0.611, Q2 = 0.352).

## Slide 4
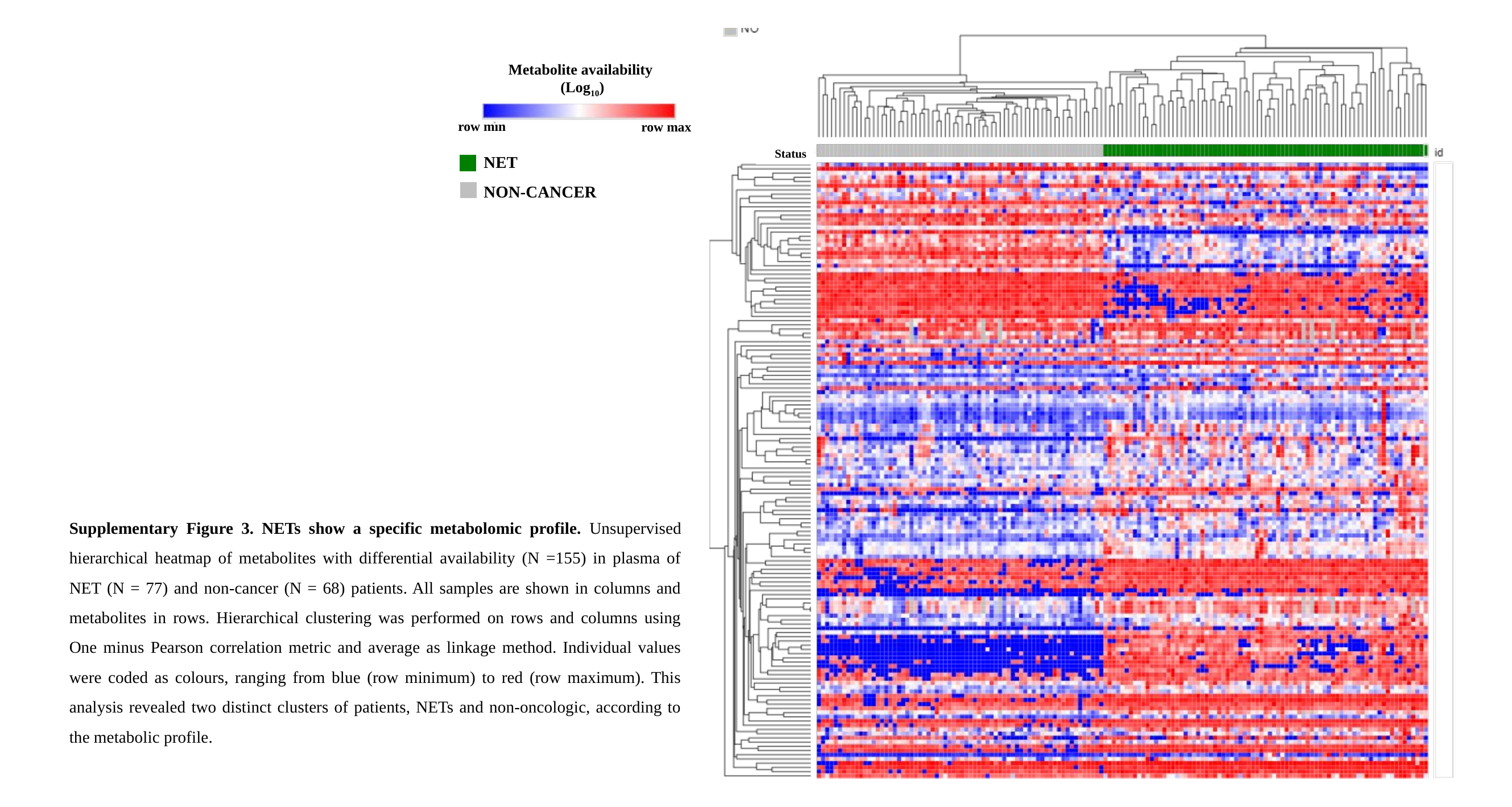

id
Metabolite availability
 (Log10)
row min
row max
NET
NON-CANCER
Status
Supplementary Figure 3. NETs show a specific metabolomic profile. Unsupervised hierarchical heatmap of metabolites with differential availability (N =155) in plasma of NET (N = 77) and non-cancer (N = 68) patients. All samples are shown in columns and metabolites in rows. Hierarchical clustering was performed on rows and columns using One minus Pearson correlation metric and average as linkage method. Individual values were coded as colours, ranging from blue (row minimum) to red (row maximum). This analysis revealed two distinct clusters of patients, NETs and non-oncologic, according to the metabolic profile.

## Slide 5
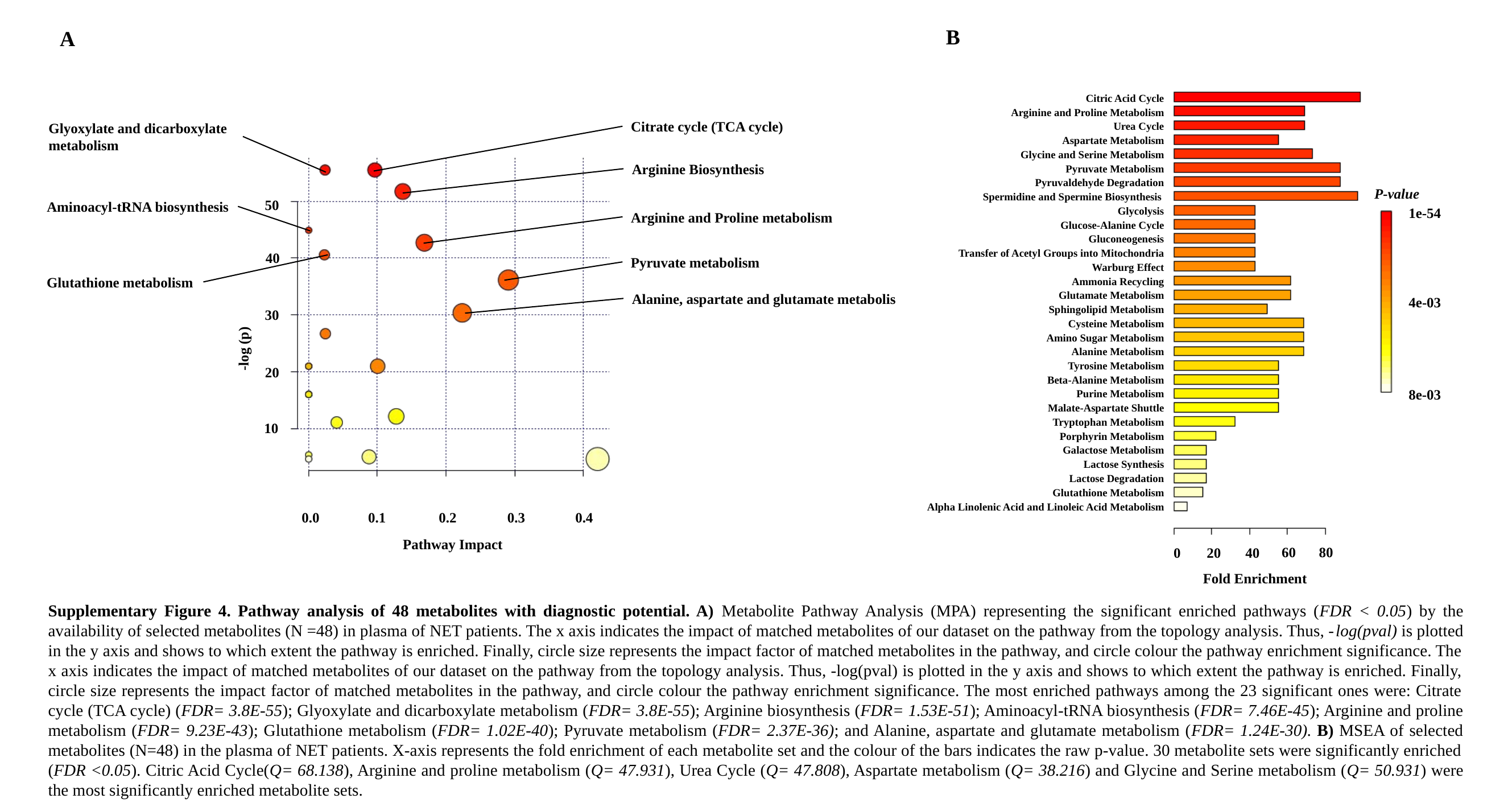

B
Citric Acid Cycle
Arginine and Proline Metabolism
Urea Cycle
Aspartate Metabolism
Glycine and Serine Metabolism
Pyruvate Metabolism
Pyruvaldehyde Degradation
Spermidine and Spermine Biosynthesis
Glycolysis
Glucose-Alanine Cycle
Gluconeogenesis
Transfer of Acetyl Groups into Mitochondria
Warburg Effect
Ammonia Recycling
Glutamate Metabolism
Sphingolipid Metabolism
Cysteine Metabolism
Amino Sugar Metabolism
Alanine Metabolism
Tyrosine Metabolism
Beta-Alanine Metabolism
Purine Metabolism
Malate-Aspartate Shuttle
Tryptophan Metabolism
Porphyrin Metabolism
Galactose Metabolism
Lactose Synthesis
Lactose Degradation
Glutathione Metabolism
Alpha Linolenic Acid and Linoleic Acid Metabolism
P-value
1e-54
4e-03
8e-03
80
60
0
40
20
Fold Enrichment
A
Citrate cycle (TCA cycle)
Glyoxylate and dicarboxylate metabolism
Arginine Biosynthesis
Aminoacyl-tRNA biosynthesis
40
Pyruvate metabolism
Glutathione metabolism
30
-log (p)
20
10
0.0
0.1
0.2
0.4
0.3
Pathway Impact
50
Arginine and Proline metabolism
Alanine, aspartate and glutamate metabolism
Supplementary Figure 4. Pathway analysis of 48 metabolites with diagnostic potential. A) Metabolite Pathway Analysis (MPA) representing the significant enriched pathways (FDR < 0.05) by the availability of selected metabolites (N =48) in plasma of NET patients. The x axis indicates the impact of matched metabolites of our dataset on the pathway from the topology analysis. Thus, -log(pval) is plotted in the y axis and shows to which extent the pathway is enriched. Finally, circle size represents the impact factor of matched metabolites in the pathway, and circle colour the pathway enrichment significance. The x axis indicates the impact of matched metabolites of our dataset on the pathway from the topology analysis. Thus, -log(pval) is plotted in the y axis and shows to which extent the pathway is enriched. Finally, circle size represents the impact factor of matched metabolites in the pathway, and circle colour the pathway enrichment significance. The most enriched pathways among the 23 significant ones were: Citrate cycle (TCA cycle) (FDR= 3.8E-55); Glyoxylate and dicarboxylate metabolism (FDR= 3.8E-55); Arginine biosynthesis (FDR= 1.53E-51); Aminoacyl-tRNA biosynthesis (FDR= 7.46E-45); Arginine and proline metabolism (FDR= 9.23E-43); Glutathione metabolism (FDR= 1.02E-40); Pyruvate metabolism (FDR= 2.37E-36); and Alanine, aspartate and glutamate metabolism (FDR= 1.24E-30). B) MSEA of selected metabolites (N=48) in the plasma of NET patients. X-axis represents the fold enrichment of each metabolite set and the colour of the bars indicates the raw p-value. 30 metabolite sets were significantly enriched (FDR <0.05). Citric Acid Cycle(Q= 68.138), Arginine and proline metabolism (Q= 47.931), Urea Cycle (Q= 47.808), Aspartate metabolism (Q= 38.216) and Glycine and Serine metabolism (Q= 50.931) were the most significantly enriched metabolite sets.

## Slide 6
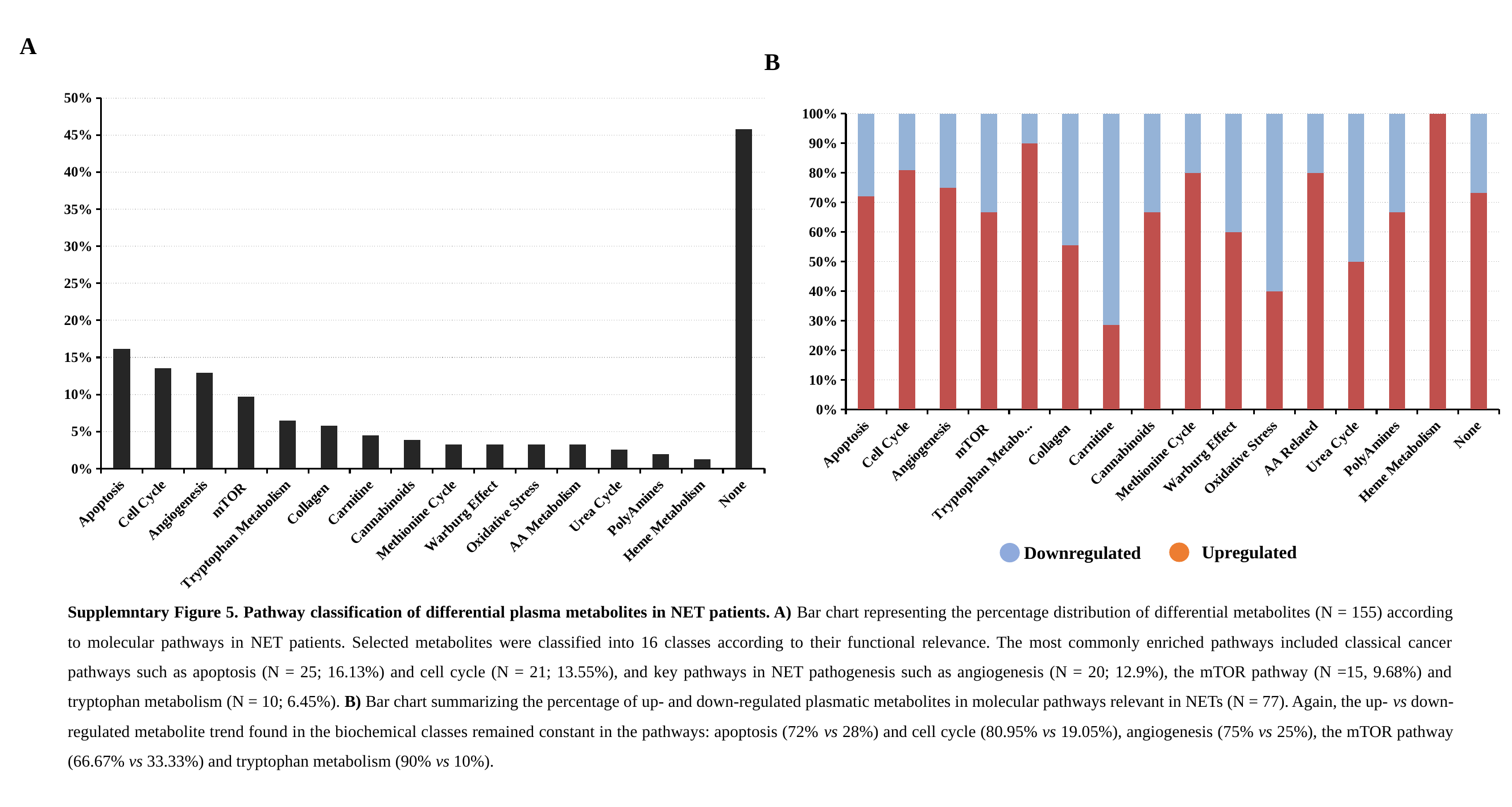

A
B
### Chart
| Category | % Total |
|---|---|
| Apoptosis | 0.16129032258064516 |
| Cell Cycle | 0.13548387096774195 |
| Angiogenesis | 0.12903225806451613 |
| mTOR | 0.0967741935483871 |
| Tryptophan Metabolism | 0.06451612903225806 |
| Collagen | 0.05806451612903226 |
| Carnitine | 0.04516129032258064 |
| Cannabinoids | 0.03870967741935484 |
| Methionine Cycle | 0.03225806451612903 |
| Warburg Effect | 0.03225806451612903 |
| Oxidative Stress | 0.03225806451612903 |
| AA Metabolism | 0.03225806451612903 |
| Urea Cycle | 0.025806451612903226 |
| PolyAmines | 0.01935483870967742 |
| Heme Metabolism | 0.012903225806451613 |
| None | 0.45806451612903226 |
### Chart
| Category | | |
|---|---|---|
| Apoptosis | 0.72 | 0.28 |
| Cell Cycle | 0.809523809523809 | 0.19047619047619 |
| Angiogenesis | 0.75 | 0.25 |
| mTOR | 0.666666666666667 | 0.333333333333333 |
| Tryptophan Metabolism | 0.9 | 0.1 |
| Collagen | 0.555555555555556 | 0.444444444444444 |
| Carnitine | 0.285714285714286 | 0.714285714285714 |
| Cannabinoids | 0.666666666666667 | 0.333333333333333 |
| Methionine Cycle | 0.8 | 0.2 |
| Warburg Effect | 0.6 | 0.4 |
| Oxidative Stress | 0.4 | 0.6 |
| AA Related | 0.8 | 0.2 |
| Urea Cycle | 0.5 | 0.5 |
| PolyAmines | 0.666666666666667 | 0.333333333333333 |
| Heme Metabolism | 1.0 | 0.0 |
| None | 0.732394366197183 | 0.267605633802817 |Upregulated
Downregulated
Supplemntary Figure 5. Pathway classification of differential plasma metabolites in NET patients. A) Bar chart representing the percentage distribution of differential metabolites (N = 155) according to molecular pathways in NET patients. Selected metabolites were classified into 16 classes according to their functional relevance. The most commonly enriched pathways included classical cancer pathways such as apoptosis (N = 25; 16.13%) and cell cycle (N = 21; 13.55%), and key pathways in NET pathogenesis such as angiogenesis (N = 20; 12.9%), the mTOR pathway (N =15, 9.68%) and tryptophan metabolism (N = 10; 6.45%). B) Bar chart summarizing the percentage of up- and down-regulated plasmatic metabolites in molecular pathways relevant in NETs (N = 77). Again, the up- vs down-regulated metabolite trend found in the biochemical classes remained constant in the pathways: apoptosis (72% vs 28%) and cell cycle (80.95% vs 19.05%), angiogenesis (75% vs 25%), the mTOR pathway (66.67% vs 33.33%) and tryptophan metabolism (90% vs 10%).
