# Supplementary material for: Comprehensive Plasma Metabolomic Profile of Patients with Advanced Neuroendocrine Tumors (NETs). Diagnostic and Biological Relevance
Source: Cancers (Basel). 2021 May 27;13(11):2634. doi: 10.3390/cancers13112634 (PMC8197817; doi:10.3390/cancers13112634)
Supplement: Supplementary file 1 [file cancers-13-02634-s001.zip › cancers-1222208-4-1supplementary/cancers-1222208-4-supplementary-tables.docx]

SUPPLEMENTARY MATERIAL AND METHODS

Comprehensive Plasma Metabolomic Profile of Patients with Advanced Neuroendocrine Tumors (NETs). Diagnostic and Biological Relevance

Beatriz Soldevilla, Angeles López-López, Alberto Lens-Pardo, Carlos Carretero-Puche, Angeles Lopez-Gonzalvez, Anna La Salvia, Beatriz Gil-Calderon, Maria C. Riesco-Martinez, Paula Espinosa-Olarte, Jacinto Sarmentero, Beatriz Rubio-Cuesta, Raúl Rincón, Coral Barbas and Rocio Garcia-Carbonero

File S1

Multiplatform metabolic fingerprinting

Plasma non-targeted analysis by CE–MS

Plasma samples (100 μL) were mixed with 100 μL of 0.2 M formic acid (with 5 % acetonitrile and 0.4 mM methionine sulfone as internal standard). Samples were vortex mixed for approximately 1 min and then transferred to a Centrifree Millipore (30 kDa) filter and centrifuged (2000 *× g*, 70 min, 4 °C). The filtered solution was transferred directly to a vial for analysis [1].

CE−MS analyses were performed using a capillary electrophoresis 7100 (Agilent Technologies, Wilmington, USA) coupled to an Accurate-Mass TOF−MS system 6224 (Agilent Technologies, Wilmington, USA). The coupling was equipped with an electrospray ionization source (ESI).

A new Agilent Technologies fused–silica capillary (50 µm i.d. x 100 cm total length) was conditioned with three stages of 30 minutes each of them: 1.0 M NaOH, followed by MiliQ® water and background electrolyte (BGE) (1.0 M formic acid in 10% Methanol). Before each analysis, the BGE vial was automatically emptied and filled. The capillary was then rinsed for 5 min (950 mbar) with BGE, applying a voltage of 30 kV for 10 s in order to displace the buffer ions. Sample injections were performed during 50 s with 50 mbar pressure. After each sample injection, the BGE was injected for 20 s at 100 mbar pressure. The separation conditions included 25 mbar of pressure and 30 kV of voltage. Data were acquired in ESI+ with a scan rate of 1.00 spectra/sec and the mass range from *m/z* 74 to 1000. The sheath liquid consisted of 50% methanol, 50 % water and 10 µL of reference standards (0.25 µM purine, (*m/z* 121.0509) + 0.25 µM HP-0921 (*m/z* 922.0098)) using a flow rate of 0.6 mL/min (1:100 split). The drying gas temperature was maintained at 200°C with a flow rate of 10 L/min; the nebulizer pressure was set to 10 psig, voltage 3,500 V, fragmentor voltage 125 V, and skimmer 65 V[1].

In-source fragmentation has been used to fragment molecules and obtain product ions to confirm the tentative annotation of the metabolite (in-source collision-induced dissociation (IS-CID)). The samples were re-analyzed using the same conditions but increasing up to 200 V the fragment voltage allowing the fragmentation of molecules in the ESI source and the mass range from m/z 50 to 1000. Thus, additional information useful to elucidate the structure with a single-stage mass analyzer was provided [2].

Plasma non-targeted analysis by LC–MS

Samples were treated for plasma deproteinization and metabolites extraction. 100 µL of plasma was mixed with 300 µL of cold (-20 °C) mixture of MeOH:EtOH (1:1, v/v). Samples were vortex-mixed for 1 min, incubated on ice for 5 min and centrifuged for 20 min at 16000 *x g* at 4 °C. The resulting supernatant was then transferred directly to a vial for analysis.

LC−MS analysis was performed on a UHPLC system 1290 Infinity II (Agilent Technologies, Waldbronn, Germany) coupled with 6545 QTOF MS detector in positive and negative ESI modes. For the separation, a volume of 0.5 µL was injected onto a Zorbax Extended-C18 Rapid Resolution column (Agilent Technologies, 2.1×50 mm, 1.8 μm) thermostated at 60 °C. The flow rate was 0.6 mL/min with a mobile phase composed of water with 0.1% formic acid for A and ACN with 0.1% formic acid for B. The chromatography gradient started from 5% B for the first min increasing to 80% B in 6.0 min, then to 100% by 11.5 min and the starting condition was returned in 0.5 min allowing re-equilibration until 15.0 min[3].

Data were collected in positive and negative ESI ionization modes in separate runs and operated in the range from *m/z* 100 to 1000, and *m/z* 40 to 1000 for MS analysis and MS/MS analysis, respectively.

The nozzle voltage was set to 1000 V, and the capillary voltage was 3000 V with a scan rate of 1.5 scans/s (positive mode) or -4000 V with a scan rate of 1.0 scan/s (negative mode). The drying gas was heated up to 250 °C and flowed at a rate of 12 L/min, pressure 52.0 psi. Additional heating was applied using sheath heated gas up to 370 ºC with a flow of 11 L/min for improving the ionization.

The MS/MS analysis was performed with the same chromatographic and spectrometric conditions used for the primary analysis. According to the prior determined accurate mass and retention time, ions of interest were targeted by collision-induced dissociation (CID) fragmentation on the fly, using a narrow isolation width (approx. 1.3 Da).

For internal mass correction during data acquisition, two reference masses were infused continuously to the system over the course of the whole analysis: *m/z* 121.0509 (protonated purine) and *m/z* 922.0098 protonated hexakis (1H,1H,3H-tetrafluoropropoxy) phosphazine (HP-921) in positive ionization mode, whereas *m/z* 112.9856 (proton-abstracted TFA anion) and *m/z* 966.0007 (formate adduct of HP921) for the negative mode.

Plasma non-targeted analysis by GC–MS

For GC–MS analysis, protein precipitation was performed by treatment with cold acetonitrile (1:3) followed by vortex-mix for 2 min and let stand on ice for 5 min. Samples were centrifuged at 15,400 x *g* for 10 min at 4 °C. For methoximation reaction, 100 μl of the supernatant were transferred to a GC vial and evaporated to complete dryness by Speedvac concentrator (SpeedVac Concentrator System, Thermo Fisher Scientific, Waltham, MA) and then, 10 µL of O-methoxyamine hydrochloride (15 mg/mL) in pyridine solution was added to the dried sample and thoroughly mixed for 1 min on a vortex mixer. Methoxymation reaction was carried out in darkness at room temperature for 16 h. For silylation process, 10 μL of BSTFA/TMCS (99:1) was added as catalyst and vortex-mixed for 5 min. Capped vials were heated in an oven for 1 h at 70 °C. Finally, 100 μL of heptane containing C18:0 methyl ester (10 mg/L) as internal standard (IS) was added to each sample and vortex-mixed prior GC analysis [4].

The analysis was performed by GC system (Agilent Technologies 7890A) coupled to a mass spectrometer with triple-Axis detector (5975C, Agilent Technologies). Two microlitres of derivatized plasma samples were automatically injected by an Agilent autosampler (7693) in split mode (split ratio 1:10) through an Agilent ultra-inert deactivated glass wool split liner. An Agilent GC column DB5-MS (30 m length, 0.25 mm i.d, and 0.25 μm film of 95% dimethyl/5% diphenylpolysiloxane) with a precolumn (10 m J&W integrated with Agilent 122-5532G) was used for compound separation. Carrier gas flow rate (He) was set approximately at 1 mL/min after performing Retention Time Locking (RTL) and, injector and transfer line temperatures at 250 °C and 280 °C, respectively. The initial column oven temperature was set at 60 °C (held for 1 minute), rose to 325 °C at 10 °C/min during 26.5 min, and hold at this temperature for 10 minutes before cooling down for the next injection. MS detection was performed with electron impact ionization (EI) with 70 eV of energy and 230 °C in filament source.

Mass spectra were collected over a mass range of m/z 50–600 at a scan rate of 2 spectra/s. Internal standard C18:0 methyl ester (10 mg/L) and, for retention index determination, a mixture of n-alkanes (C8-C28) dissolved in n-hexane were injected prior to the samples. Data were acquired using Agilent MSD ChemStation Software (Agilent Technologies).

The analysis was carried out by randomising the samples for each platform run. For equipment performance and reproducibility determination, several replicates were analysed from a homogeneous pool containing a small equal amount of all the samples (Quality Controls, QC). These QCs were treated like the rest of the samples. They were injected at the beginning of the batch (10 injections) to equilibrate the system and every ten samples to monitor the stability of the analysis[5].

Data Processing

The raw data obtained by CE−MS were processed with MassHunter Profinder software version B.08.00, applying the Molecular Feature Extraction (MFE) to clean off background noise and unrelated ions. Coeluting adducts of the same feature (+H^+^, +Na^+^, and neutral loss of water) were searching by MFE where all the features (ions) were aligned across the samples using mass and retention time (RT). Therefore, a final spectrum for each compound group was built to continue with the next step, the reextraction of the batch files. Batch Recursive Feature Extraction (RFE) refines the quality of the target list for the Find by Ion (FbI) function by improving the quality of the final list of compounds. By RFE, using both the mass and the RT of the previous MFE results, a final compound group list was generated, and the verification of the correct integration of extracted ion chromatogram was inspected for each feature, through all the samples, one by one.

The raw data collected by LC−MS were reprocessed by the molecular feature extraction (MFE) with Mass Hunter Qualitative (B.06.00, Agilent Software). The MFE algorithm enables us to clean data background noise and creates a final list of possible components. As a way of finding coeluting adducts of the same feature, data were reprocessed using DA Reprocessor Offline Utilities B.05.00 (Agilent) for ions such as [M]^+^, [M+H]^+^, [M+Na]^+^ in positive ionization, [M−H]^-^, [M+HCOOH-H]^−^, [M+Cl]^-^ in negative ion mode, and neutral loss of water in both polarities. Data were aligned and filtered using Mass Profiler Professional software (B.14.9 Agilent Software).

GC−MS data treatment started with a thorough inspection of the total ion chromatograms (TIC) of experimental samples with examination of the overall quality of analytical performance. Raw data files were converted to the appropriate format for quantitative analysis through MassHunter Workstation GC-MS Translator (B.04.01). Agilent MassHunter Unknowns Analysis Tool 7.0 was used for deconvolution and metabolite identification. The assignment of a chemical identity was done by searching via two specific libraries: Fiehn, 2008 version, and “in house” plasma spectral library from CEMBIO based on Fiehn and NIST (National Institute of Standards and Technology, library 2.2 version 2014) libraries. This identity was granted comparing their retention indices and retention times, and spectrum extracted after deconvolution with each compound included in the libraries. Afterward the data was aligned with MassProfiler Professional (B.14.9 Agilent Software) and exported to Agilent MassHunter Quantitative Analysis (B.07.00) for target ion assignation and obtaining the abundance of compounds with the inspection of the correct integration of peaks. Finally, the data matrix with the final abundance of each metabolite was generated.

To ensure valid measurements, data matrix obtained after data reprocessing for each platform were filtered according to the variation in the abundance of the compounds in QC samples, expressed as standard deviation relative, and only those with RSD<30% were kept.

Statistical analysis

The three hyperparameters that determine the accuracy of the within-batch effects elimination were: i) the tolerance threshold (ε) 5% of the median of distribution of QC values, ii) the penalty term (C) median of the distribution of QC samples and iii) the kernel width (γ) logspace (0,3) 20 values.

OPLS-DA models were validated for every platform using response of permutation test through 999 permutations. This test assesses whether the specific classification of individuals in the two designed groups is significantly better than any other random classification into two arbitrary groups. Permutation test shows the validity and degree of overfitting for model. The R^2^ and Q^2^ distribution is compared to the original (unperturbed)) data when the Y data is randomly permutated. At the same time, the X data is left intact. The resulting validation plot represents on the y-axis the R^2^ and Q^2^ values of the original and permuted models, while the x-axis represents the correlation coefficients between both models. The points were fitted via the regression line. Model validity should have higher R^2^ and Q^2^ values of the original models than of the permutated models [6].

Annotation and compound identification

An initial tentative identification of features from LC−MS and CE−MS based on the *m/z* of the compounds showing significant differences in class separation was performed by CEU Mass Mediator tool[7]. Tentative annotation covered, beside the accurate mass matching with the mass error set to 10 ppm for LC−MS and 20 ppm for CE−MS, isotopic distribution determination and manual checking of the possible ions and adducts. For CE−MS, fragments, dimers or ringing artefacts were removed from the dataset as described in Godzien et al.[8]

Spectra at high fragmentor voltage (200 V) were used to confirm CE−MS annotations. As a result, fragmentation spectra were obtained and ion-source fragmentation characteristic patterns were studied. For this purpose, an in-house database created by CEMBIO of 515 fragments has been used together with its ions, adducts and multimers, which is available in CEU Mass Mediator (CMM) [7,9] and includes a relative migration time library.

To confirm the annotation of the compounds, LC−MS/MS analysis was carried out, repeating the experiment. The data independent analysis (DIA) was performed with the same chromatographic and spectrometric conditions used for the primary analysis. According to the prior determined accurate mass and retention time, ions of interest were targeted by collision-induced dissociation (CID) fragmentation, using a narrow isolation width (approx. 1.3 Da). Precursor ions were targeted with different fixed collision energies at 20 and 40eV, for separate runs to obtain collision energy-specific MS/MS spectra.

Targeted analysis

HPLC QqQ MS/MS method optimization

After LC-MS optimization, the binary mobile phase selected consisted of phase A (0.1% FA in water) and phase B (0.1% FA and 10% iPrOH in ACN). The flow used was adjusted to 0.6 mL/min, and the injection volume was 1.00 µL for all standards and samples studied. The chromatography gradient started with 3% phase B, increasing to 4% B at minute 0.75. The gradient then grew to 45% B at minute 0.85 and held until minute 1.80. Another increment was applied to 79% B at minute 1.90, maintaining this percentage until minute 3.40. In 0.10 minutes, it rose to 100% B and was maintained until minute 5.00. Finally, the starting condition was returned in 0.10 min allowing re-equilibration until 6.0 min. Mass spectral data acquisition was achieved under positive (ESI +) and negative polarity (ESI −) according to the metabolite and dynamic multiple reaction monitoring (dMRM) mode. The capillary voltage was set to 4000 V for positive and 3500 V for negative ionization mode; the drying gas flow rate was 8 L/min at 200 °C, the gas nebulizer at 50 psi, sheath gas flow 8L/min, and sheath gas temperature 250 °C.

Reference

1. Naz, S.; Garcia, A.; Rusak, M.; Barbas, C. Method Development and Validation for Rat Serum Fingerprinting with CE-MS: Application to Ventilator-Induced-Lung-Injury Study. *Anal. Bioanal. Chem.* 2013, *405*, 4849–4858, doi:10.1007/s00216-013-6882-5.
2. Mamani-Huanca, M.; Gradillas, A.; Gil de la Fuente, A.; López-Gonzálvez, Á.; Barbas, C. Unveiling the Fragmentation Mechanisms of Modified Amino Acids as the Key for Their Targeted Identification. *Anal. Chem.* 2020, *92*, 4848–4857, doi:10.1021/acs.analchem.9b04313.
3. Godzien, J.; Kalaska, B.; Adamska-Patruno, E.; Siroka, J.; Ciborowski, M.; Kretowski, A.; Barbas, C. Oxidized Glycerophosphatidylcholines in Diabetes through Non-Targeted Metabolomics: Their Annotation and Biological Meaning. *J. Chromatogr. B Analyt. Technol. Biomed. Life. Sci.* 2019, *1120*, 62–70, doi:10.1016/j.jchromb.2019.04.053.
4. Dudzik, D.; Zorawski, M.; Skotnicki, M.; Zarzycki, W.; Kozlowska, G.; Bibik-Malinowska, K.; Vallejo, M.; García, A.; Barbas, C.; Ramos, M.P. Metabolic Fingerprint of Gestational Diabetes Mellitus. *J. Proteomics* 2014, *103*, 57–71, doi:10.1016/j.jprot.2014.03.025.
5. Dudzik, D.; Barbas-Bernardos, C.; García, A.; Barbas, C. Quality Assurance Procedures for Mass Spectrometry Untargeted Metabolomics. a Review. *J. Pharm. Biomed. Anal.* 2018, *147*, 149–173, doi:10.1016/j.jpba.2017.07.044.
6. Barnes, R.J.; Dhanoa, M.S.; Lister, S.J. Standard Normal Variate Transformation and De-Trending of Near-Infrared Diffuse Reflectance Spectra. *Appl. Spectrosc.* 1989, *43*, 772–777.
7. Gil de la Fuente, A.; Godzien, J.; Fernández López, M.; Rupérez, F.J.; Barbas, C.; Otero, A. Knowledge-Based Metabolite Annotation Tool: CEU Mass Mediator. *J. Pharm. Biomed. Anal.* 2018, *154*, 138–149, doi:10.1016/j.jpba.2018.02.046.
8. Godzien, J.; Armitage, E.G.; Angulo, S.; Martinez-Alcazar, M.P.; Alonso-Herranz, V.; Otero, A.; Lopez-Gonzalvez, A.; Barbas, C. In-Source Fragmentation and Correlation Analysis as Tools for Metabolite Identification Exemplified with CE-TOF Untargeted Metabolomics. *Electrophoresis* 2015, *36*, 2188–2195, doi:10.1002/elps.201500016.
9. Gil-de-la-Fuente, A.; Godzien, J.; Saugar, S.; Garcia-Carmona, R.; Badran, H.; Wishart, D.S.; Barbas, C.; Otero, A. CEU Mass Mediator 3.0: A Metabolite Annotation Tool. *J. Proteome Res.* 2019, *18*, 797–802, doi:10.1021/acs.jproteome.8b00720.

**Table S1.** Clinical, biochemical and pathological features of NET population.

| Features. | N (%) |
| --- | --- |
| **Age (years)** |  |
| Median value (range) | 63 (37-83) |
| **BMI** |  |
| Median value (range) | 25.9 (17.2- 52.5) |
| **Gender** |  |
| Female | 35 (45.5%) |
| Male | 42 (54.5%) |
| **Localization of primary tumor** |  |
| Lung | 18 (23.4%) |
| Small Intestine | 45 (58.4%) |
| Colon & Rectum | 8 (10.4%) |
| Others | 6 (7.8%) |
| **Grade** |  |
| G1 | 26 (33.8%) |
| G2 | 51 (66.2%) |
| **Ki67 (%)** |  |
| <5 | 38 (49.3%) |
| 5-9 | 24 (31.1%) |
| 10-20 | 15 (19.4%) |
| **Functioning Tumor** |  |
| Yes (Carcinoid Syndrome) | 24 (31.2%) |
| No | 53 (68.8%) |
| **Time from diagnosis to study entry** |  |
| < 12 months | 34 (44.2%) |
| ≥ 12 months | 41 (53.2%) |
| Unknown | 2 (2.6%) |
| **Cromogranin A** |  |
| % > ULN | 57 (74.0%) |
| % > 2x ULN | 51 (66.2%) |
| **5-HIAA** |  |
| % > ULN | 46 (59.7%) |
| % > 2x ULN | 39 (50.6%) |
| **LDH > ULN** |  |
| Yes | 7 (9.1%) |
| No | 23 (29.9%) |
| Unknown | 47 (61.0%) |
| **Alkaline Phosphatase >ULN** |  |
| Yes | 6 (7.8%) |
| No | 15 (19.5%) |
| Unknown | 56 (72.7%) |
| **AST > ULN** |  |
| Yes | 9 (11.7%) |
| No | 68 (88.3%) |
| **ALT > ULN** |  |
| Yes | 7 (9.1%) |
| No | 70 (90.9%) |
| **Glycemia > ULN** |  |
| Yes | 25 (32.5%) |
| No | 51 (66.2%) |
| Unknown | 1 (1.3) |
| **Creatinine > ULN** |  |
| Yes | 8 (10.4%) |
| No | 69 (89.6%) |
| **Urea > ULN** |  |
| Yes | 11 (14.3%) |
| No | 65 (84.4%) |
| Unknown | 1 (1.3%) |

ULN: Upper Limit of Normal.

**Table S2.** Metabolites with biomarker potential in NET patients. This table summarizes the ROC and OPLS-DA analyses of the 155 differential plasmatic metabolites identified between NET (N = 77) and non-cancer (N = 68) patients. *AUC*, sensitivity and specificity (according to Youden Index) are shown for each metabolite. *p(corr)* and *VIP* (*PLS-DA* parameters) are also displayed for significant metabolites. Those with an *AUC > 0.85,* a *VIP > 1.0* and a *|p(corr)| > 0.5* or both were considered as metabolites with biomarker potential. 27 metabolites had both an *AUC > 0.85*. and a *VIP > 1.0* and a *|p(corr)| > 0.5*; 17 metabolites had only a *VIP > 1.0* and a *|p(corr)| > 0.5* and 5 metabolites had only an *AUC > 0.85*. Overall, 49 metabolites showed biomarker potential. (* = multiple identification options).

| **Metabolite** | **AUC** | **Specificity** | **Sensitivity** | **p(corr)** | **VIP** |
| --- | --- | --- | --- | --- | --- |
| Lactic acid | 0.999 | 0.985 | 1.000 | -0.918 | 2.576 |
| (Homo)2-aconitate* | 0.998 | 1.000 | 0.987 | 0.843 | 2.984 |
| Succinylacetoacetate* | 0.998 | 1.000 | 0.987 | 0.843 | 2.984 |
| Arginine | 0.982 | 0.956 | 0.961 | -0.795 | 2.326 |
| Biliverdin | 0.962 | 0.941 | 0.896 | -0.724 | 2.620 |
| Anthraniloyl-CoA | 0.954 | 0.941 | 0.896 | -0.689 | 2.387 |
| Stearoyl-tyrosine* | 0.938 | 0.794 | 0.948 | 0.755 | 3.742 |
| Arachidonoylcarnitine* | 0.938 | 0.794 | 0.948 | 0.755 | 3.742 |
| 3-hydroxy-5-octenoylcarnitine | 0.937 | 0.971 | 0.883 | -0.723 | 3.964 |
| Lynoleilcarnitine | 0.937 | 0.941 | 0.805 | 0.732 | 3.395 |
| Pyruvic acid | 0.904 | 0.892 | 0.795 | -0.686 | 1.784 |
| Ornithine | 0.899 | 0.926 | 0.779 | 0.646 | 1.654 |
| PYRANOSE (glucose/altrose /galactose /talose ) | 0.899 | 0.831 | 0.849 | 0.633 | 1.713 |
| 5-Hydroxyindoleacetic acid | 0.891 | 0.985 | 0.792 |  |  |
| Suberylglycine | 0.890 | 1.000 | 0.779 | -0.734 | 3.819 |
| 4-Hydroxycyclohexylcarboxylic acid | 0.888 | 0.794 | 0.909 | -0.713 | 2.992 |
| 3-Hydroxydodecanoic acid | 0.887 | 0.809 | 0.922 | -0.725 | 2.793 |
| Glucose | 0.887 | 0.862 | 0.808 | 0.620 | 1.636 |
| Trp-Phe | 0.887 | 0.765 | 0.896 | -0.626 | 1.555 |
| Allose | 0.880 | 0.815 | 0.863 | 0.618 | 1.437 |
| Glu-Arg | 0.879 | 0.882 | 0.779 | -0.524 | 1.548 |
| 3-Hydroxydodecanedioic acid | 0.876 | 0.809 | 0.870 | -0.679 | 2.975 |
| MG(20:0) | 0.873 | 0.868 | 0.896 |  |  |
| Aspartate | 0.872 | 0.912 | 0.766 |  |  |
| PG(28:0) | 0.871 | 0.824 | 0.779 | -0.605 | 2.750 |
| Bilirubin | 0.861 | 0.926 | 0.779 | -0.679 | 2.628 |
| Cholestane-3.7.12.24.25-pentol | 0.858 | 0.794 | 0.818 | 0.555 | 2.034 |
| 1-Methyladenosine | 0.857 | 0.779 | 0.831 | -0.580 | 1.198 |
| Glu-Ala* | 0.853 | 0.926 | 0.753 |  |  |
| Ser-hyp* | 0.853 | 0.926 | 0.753 |  |  |
| Dodecenedioic acid | 0.852 | 0.809 | 0.779 | -0.600 | 2.237 |
| Indoleacetyl glutamine | 0.852 | 0.824 | 0.766 | -0.580 | 2.047 |
| PG(20:2) | 0.846 | 0.985 | 0.636 | 0.587 | 2.332 |
| HETE | 0.846 | 0.809 | 0.805 | -0.630 | 2.642 |
| Cys-Gly disulfide | 0.837 | 0.794 | 0.792 | 0.493 | 1.630 |
| Linolenyl carnitine | 0.834 | 0.824 | 0.831 | 0.534 | 2.261 |
| Calcitroic acid | 0.830 | 0.632 | 0.896 | -0.536 | 1.759 |
| Oleoylcarnitine/Elaidic carnitine | 0.828 | 0.765 | 0.818 | 0.511 | 2.402 |
| Docosapentaenoic acid | 0.824 | 0.824 | 0.740 | -0.550 | 1.513 |
| Eicosenoic acid | 0.822 | 0.662 | 0.831 | -0.511 | 1.495 |
| Glu-Lys/Ɛ-Glu-Lys | 0.821 | 0.926 | 0.636 |  |  |
| Leu-Phe | 0.820 | 0.882 | 0.740 | -0.601 | 2.449 |
| 5-Hydroxyindoleacetaldehyde | 0.817 | 0.632 | 0.870 | -0.512 | 2.553 |
| Eicosatrienoic acid | 0.813 | 0.691 | 0.805 |  |  |
| Serotonine | 0.810 | 0.574 | 0.974 |  |  |
| LPC(18:2)-OH | 0.810 | 0.971 | 0.649 |  |  |
| Ursodeoxycholic acid 3-sulfate | 0.807 | 0.662 | 0.922 | -0.544 | 2.357 |
| Methionine S-oxide | 0.806 | 0.838 | 0.688 |  |  |
| 12a-Hydroxy-3-oxocholadienic acid | 0.804 | 0.721 | 0.844 | -0.537 | 2.119 |
| Sphingosine-1-phosphate | 0.803 | 0.838 | 0.675 | 0.502 | 2.058 |
| Dehydroepiandrosterone 3-glucuronide /Dehydroisoandrosterone 3-glucuronide | 0.799 | 0.647 | 0.792 |  |  |
| Phenylglucuronide | 0.794 | 0.853 | 0.740 | -0.524 | 2.375 |
| beta-Phenylalanoyl-CoA* | 0.789 | 0.779 | 0.753 |  |  |
| LPE(20:5) | 0.784 | 0.735 | 0.870 |  |  |
| Thr-Ala | 0.780 | 0.926 | 0.623 |  |  |
| ecdysone 25-O-D-glucopyranoside | 0.780 | 0.662 | 0.792 |  |  |
| Ursodeoxycholic acid | 0.779 | 0.765 | 0.818 | -0.510 | 2.349 |
| LPA(13:0) | 0.779 | 0.971 | 0.714 | 0.556 | 2.286 |
| Cys-Gly | 0.775 | 0.809 | 0.636 |  |  |
| 3-Indoleacetic acid | 0.772 | 0.738 | 0.753 |  |  |
| Leu-hyp | 0.772 | 0.691 | 0.805 |  |  |
| PS(39:5) | 0.768 | 0.941 | 0.481 |  |  |
| beta-Phenylalanoyl-CoA* | 0.765 | 0.500 | 0.922 |  |  |
| LPC(22:1) | 0.763 | 0.750 | 0.740 |  |  |
| Cer(35:0) | 0.761 | 0.941 | 0.442 |  |  |
| Mandelic acid | 0.760 | 0.706 | 0.792 |  |  |
| 24-Hydroxygeminivitamin D3 | 0.758 | 0.706 | 0.740 |  |  |
| Arachidonic acid | 0.757 | 0.735 | 0.688 |  |  |
| N6-Acetyl-hydroxy-lysine* | 0.757 | 0.897 | 0.584 |  |  |
| Ser-Val* | 0.757 | 0.897 | 0.584 |  |  |
| Hypoxanthine | 0.756 | 0.853 | 0.597 |  |  |
| PC(32:0) | 0.754 | 0.544 | 0.844 |  |  |
| LPI(16:1) | 0.754 | 0.544 | 0.935 |  |  |
| 11-Oxo-androsterone glucuronide | 0.753 | 0.956 | 0.584 | 0.533 | 2.064 |
| Ser-Ala* | 0.753 | 0.691 | 0.740 |  |  |
| Thr-Gly* | 0.753 | 0.691 | 0.740 |  |  |
| Glu-Lys/Ɛ-Glu-Lys | 0.750 | 0.912 | 0.584 |  |  |
| Cholestane-3.7.12.25-tetrol-3-glucuronide | 0.750 | 0.868 | 0.506 |  |  |
| Pregnanediol | 0.747 | 0.971 | 0.468 |  |  |
| 4-Methylcatechol | 0.745 | 0.971 | 0.442 |  |  |
| Eicosapentaenoic acid | 0.737 | 0.750 | 0.688 |  |  |
| Glycerol | 0.735 | 0.615 | 0.753 |  |  |
| Quinoline | 0.732 | 0.750 | 0.688 |  |  |
| C27 bile acid (Hydroxy-3-oxo-4-cholestenoate) | 0.732 | 0.882 | 0.610 |  |  |
| Cysteineglutathione disulfide | 0.731 | 0.691 | 0.740 |  |  |
| 8-amino-7-oxo-nonanoic acid* | 0.730 | 0.912 | 0.558 |  |  |
| Glu-hyp | 0.729 | 0.941 | 0.455 |  |  |
| Cortisone acetate | 0.724 | 0.662 | 0.714 |  |  |
| Acetylspermidine | 0.718 | 0.618 | 0.753 |  |  |
| DG(31:0) | 0.716 | 0.765 | 0.662 |  |  |
| LPS(18:0) | 0.713 | 0.838 | 0.636 |  |  |
| Edetic Acid | 0.712 | 0.765 | 0.623 |  |  |
| Methylimidazole | 0.710 | 0.662 | 0.714 |  |  |
| N2-Methyl-lysine | 0.709 | 0.632 | 0.896 |  |  |
| Glucosylgalactosylhydroxylysine | 0.706 | 0.618 | 0.740 |  |  |
| Pyroglutamine | 0.706 | 0.750 | 0.636 |  |  |
| Isocitric acid/Citric acid | 0.704 | 0.956 | 0.377 |  |  |
| PC(38:5) | 0.699 | 0.735 | 0.623 |  |  |
| Retinol | 0.698 | 0.721 | 0.597 |  |  |
| N-(4-Coumaroyl)-homoserine lactone | 0.697 | 0.721 | 0.610 |  |  |
| LPE(22:6) | 0.697 | 0.632 | 0.714 |  |  |
| Urocanate / NicotinamideN-oxide | 0.695 | 0.706 | 0.610 |  |  |
| LPC(16:0)-OH | 0.693 | 0.618 | 0.766 |  |  |
| Dimethyl-Arginine (symmetric) | 0.693 | 0.721 | 0.675 |  |  |
| MG(18:2) | 0.693 | 0.706 | 0.753 |  |  |
| Glutamine | 0.693 | 0.779 | 0.584 |  |  |
| LPE(20:5) | 0.689 | 0.765 | 0.571 |  |  |
| p-Phenolsulfonic acid | 0.689 | 0.956 | 0.351 |  |  |
| Cer(36:1) | 0.688 | 0.750 | 0.597 |  |  |
| PC(38:2) | 0.688 | 0.647 | 0.675 |  |  |
| N-palmitoyl glutamic acid* | 0.688 | 0.721 | 0.636 |  |  |
| 3-Hydroxy-5-tetradecenoylcarnitine* | 0.688 | 0.721 | 0.636 |  |  |
| PE(34:2)/PE(O-34:3) | 0.687 | 0.838 | 0.506 |  |  |
| PE(38:6) | 0.685 | 0.853 | 0.519 |  |  |
| LPC(16:0)-OH | 0.684 | 0.721 | 0.636 |  |  |
| Arg-Val | 0.674 | 0.382 | 0.987 |  |  |
| N-acetyl-lysine | 0.673 | 0.662 | 0.636 |  |  |
| GalactosylhydroxyLys | 0.670 | 0.324 | 0.935 |  |  |
| SM(36:0) | 0.668 | 0.779 | 0.532 |  |  |
| LPE(22:6) | 0.667 | 0.794 | 0.519 |  |  |
| Chenodeoxycholic acid 3-glucuronide* | 0.663 | 0.794 | 0.506 |  |  |
| Deoxycholic acid 3-glucuronide* | 0.663 | 0.794 | 0.506 |  |  |
| Oleic acid | 0.660 | 0.846 | 0.493 |  |  |
| {[(2E)-3-phenylprop-2-en-1-yl]oxy}sulfonic acid | 0.659 | 0.853 | 0.506 |  |  |
| 8-Hydroxycarteolol | 0.657 | 0.912 | 0.494 |  |  |
| Mannitol | 0.655 | 0.769 | 0.534 |  |  |
| 3-carboxy-4-methyl-5-propyl-2-furanpropanoic acid (CMPF) | 0.654 | 0.706 | 0.558 |  |  |
| Pregnanolone sulfate | 0.652 | 0.456 | 0.857 |  |  |
| Homocitrulline | 0.650 | 0.456 | 0.818 |  |  |
| LPC(18:0)-OH | 0.649 | 0.706 | 0.623 |  |  |
| Iminodiacetic acid | 0.642 | 0.662 | 0.616 |  |  |
| 5.6-Dihydrothymine | 0.641 | 0.441 | 0.818 |  |  |
| N2-Methylproline | 0.638 | 0.662 | 0.649 |  |  |
| Glu-Asp | 0.638 | 0.706 | 0.649 |  |  |
| LPE(16:0) | 0.638 | 0.794 | 0.481 |  |  |
| Norcotinine | 0.636 | 0.529 | 0.714 |  |  |
| Phenylalanine | 0.636 | 0.544 | 0.701 |  |  |
| Phosphocholine | 0.634 | 0.382 | 0.922 |  |  |
| Val-Leu | 0.629 | 0.926 | 0.325 |  |  |
| Glu-Val | 0.627 | 0.956 | 0.390 |  |  |
| Triethylamine | 0.615 | 0.265 | 0.974 |  |  |
| Vaccenic acid | 0.613 | 0.477 | 0.726 |  |  |
| gamma-Glu-orn * | 0.613 | 0.868 | 0.442 |  |  |
| Lys-Asp* | 0.613 | 0.868 | 0.442 |  |  |
| 1-Aminocyclohexanecarboxylic acid | 0.607 | 0.529 | 0.701 |  |  |
| LPE(P-16:0) | 0.605 | 0.941 | 0.403 |  |  |
| 9-Decenoylcarnitine | 0.601 | 0.868 | 0.338 |  |  |
| di-Hydroxymelatonin* | 0.595 | 0.794 | 0.429 |  |  |
| Acetyl-N-formyl-5-methoxykynurenamine (AFMK)* | 0.595 | 0.794 | 0.429 |  |  |
| Piperideine | 0.594 | 0.809 | 0.390 |  |  |
| Pipecolic acid | 0.589 | 0.492 | 0.685 |  |  |
| Indoxylsulfuric acid | 0.582 | 0.985 | 0.221 |  |  |
| 5-Hydroxyindole | 0.578 | 0.868 | 0.338 |  |  |
| Gly-Pro | 0.562 | 0.956 | 0.260 |  |  |
| Glycine | 0.559 | 0.215 | 0.973 |  |  |

**Table S4.** Logistic regression analysis of metabolites with biomarker potential in the plasma of NET patients. A LRM model was built for each metabolite with biomarker potential. Each model was adjusted with clinical covariates (sex, age, glycaemic levels and creatinine levels) and with up to 2 significantly associated drug intake covariates in order to assess the independent diagnostic ability of each metabolite. Clinical and drug intakes covariates included in each model are indicated in the model adjustment. Exp (B) and its 95% CI and the *P-value* of each metabolite in their model is shown. *P-values* < 0.05 were considered significant. All metabolites but suberylglicine and lactate independently discriminated NET patients from non-oncologic individuals.

| **Model adjustment** | **Metabolite** | |
| --- | --- | --- |
|  | **Exp (B) 95% CI** | ***P-value*** |
| 1-methyladenosine, AGE, SEX, GLU, CRE, DIU, APAP | 2.63∙10^7^ (4.49∙10^4^ – 1.55∙10^10^) | 1.49∙10^-7^ |
| 11-Oxo-androsterone glucuronide, AGE, SEX, GLU, CRE | 0.06 (0.01 – 0.59) | 1.49∙10^-2^ |
| 12a-Hydroxy-3-oxocholadienic acid, AGE, SEX, GLU, CRE, RAN | 22.45 (4.67 – 108.01) | 1.04∙10^-4^ |
| 3-hydroxy-5-octenoylcarnitine, AGE, SEX, GLU, CRE, RAN, STE | 3.13 (2.15 – 4.55) | 2.13∙10^-9^ |
| 3-Hydroxydodecanedioic acid, AGE, SEX, GLU, CRE | 2.31 (1.82 – 2.94) | 8.84∙10^-12^ |
| 3-Hydroxydodecanoic acid, AGE, SEX, GLU, CRE | 3.04 (1.56 – 1.82∙10^3^) | 3.07∙10^-12^ |
| 4-Hydroxycyclohexylcarboxylic acid, AGE, SEX, GLU, CRE | 2.53 (1.93 – 3.30) | 1.25∙10^-11^ |
| 5-Hydroxyindoleacetaldehyde, AGE, SEX, GLU, CRE, RAN, STE | 77.24 (11.80 – 505.37) | 6.00∙10^-6^ |
| 5-Hydroxyindoleacetic acid, AGE, SEX, GLU, CRE, RAN, APAP | 3.83 (2.13 – 6.87) | 7.00∙10^-6^ |
| Allose, AGE. SEX. GLU, CRE, APT, | 11.78 (3.15 – 44.09) | 2.50∙10^-4^ |
| Anthraniloyl-CoA, AGE, SEX, GLU, CRE | 2.51∙10^8^ (1.39∙10^5^ – 4.54∙10^11^) | 4.32∙10^-7^ |
| Arachidonoylcarnitine*, AGE. SEX. GLU, CRE, APT, AHT | 1.10∙10^-5^ (1.79∙10^-7^ - 7.02∙10^-4^) | 6.66∙10^-8^ |
| Aspartate, AGE. SEX. GLU, CRE, RAN, STAT | 4.31∙10^-4^ (2.10∙10^-5^ - 9.07∙10^-3^) | 6.17∙10^-7^ |
| Arginine, AGE, SEX, GLU, CRE, RAN | 2.70∙10^7^ (1.80∙10^4^ - 4.05∙10^10^) | 4.00∙10^-6^ |
| Bilirubin, AGE, SEX, GLU, CRE, STE | 2.30 (1.78 – 2.97) | 2.21∙10^-10^ |
| Biliverdin, AGE, SEX, GLU, CRE, RAN, AHT | 3.27∙10^5^ (2.39∙10^3^ – 4.49∙10^7^ | 4.25∙10^-7^ |
| Calcitroic acid, AGE, SEX, GLU, CRE, NSAIDs, AHT | 5.39∙10^3^ (139.60 – 2.08∙10^5^) | 4.00∙10^-6^ |
| Cholestane-3,7,12,24,25-pentol, AGE, SEX, GLU, CRE | 3,04∙10^-3^ (3.32∙10^-3^ – 0.28) | 2.91∙10^-7^ |
| Cys-Gly disulfide, AGE. SEX. GLU, CRE | 1.70∙10^-5^ (2.22∙10^-7^ - 1.29∙10^-3^) | 6.65∙10^-7^ |
| Docosapentaenoic acid, AGE. SEX. GLU, CRE, STE, AHT | 1.13∙10^4^ (340.9 - 3.73∙10^5^) | 1.73∙10^-7^ |
| Dodecenedioic acid, AGE. SEX. GLU, CRE | 150.65 (18.62 – 1.21∙10^3)^ | 3.00∙10^-6^ |
| Eicosenoic acid, AGE, SEX, GLU, CRE, RAN | 2.59∙10^3^ (111.99 – 5.97∙10^4^) | 9.30∙10^-7^ |
| Glu-Ala*, AGE. SEX. GLU, CRE, RAN, STAT | 5.60∙10^3^ (1.92∙10^2^ - 1.64∙10^5^) | 5.35∙10^-7^ |
| Glu-Arg, AGE. SEX. GLU, CRE, | 1.52∙10^4^ (5.22∙10^2^ - 4.43∙10^5^) | 2.19∙10^-8^ |
| Glucose, AGE, SEX, GLU, CRE, APT, RAN | 10.75 (3.16 – 36.52) | 1.42∙10^-4^ |
| HETE, AGE. SEX. GLU, CRE, PPI, AHT | 2.00 (1.60 – 2.50) | 2.22∙10^-9^ |
| Homo 2-aconitate*, AGE, SEX, GLU, CRE, RAN, AHT | 1.33∙10^-17^ (0 - 0.52) | 4.62∙10^-2^ |
| Indoleacetyl glutamine, AGE. SEX. GLU, CRE, RAN | 61.80 (9.72 - 392.56) | 1.20∙10^-5^ |
| Lactate, AGE, SEX, GLU, CRE, RAN, AHT | 4.50∙10^-25^ (0 - 6.33) | 5.78∙10^-2^ |
| Leu-Phe, AGE. SEX. GLU, CRE, RAN, AHT | 2.44 (1.81 – 3.27) | 3.69∙10^-9^ |
| Linolenyl carnitine, AGE, SEX, GLU, CRE, STE | 3.50∙10^-4^ (1.71∙10^-3^ – 7.19∙10^-5^) | 2.45∙10^-7^ |
| Linoleyl carnitine, AGE, SEX, GLU, CRE, STE, RAN | 4.00∙10^-6^ (4.57∙10^-8^ – 3.45∙10^-4^) | 4.80∙10^-8^ |
| LPA (13:0), AGE, SEX, GLU, CRE, RAN | 0.29 (0.17 – 0.48) | 2.00∙10^-6^ |
| MG (20:00), AGE, SEX, GLU, CRE, RAN | 0.39 (0.30 – 0.51) | 5.47∙10^-12^ |
| Ornithine, AGE. SEX. GLU, CRE | 1.73∙10^-7^ (1.09∙10^-9^ - 2.74∙10^-5^) | 1.71∙10^-9^ |
| PG (20:2), AGE, SEX, GLU, CRE | 1.00∙10^-3^ (5.50∙10^-5^ – 0.02) | 3.00∙10^-6^ |
| PG (28:0), AGE, SEX, GLU, CRE, APT, DIU | 2.36∙10^3^ (1.28∙10^2^ – 4.37∙10^4^) | 1.82∙10^-7^ |
| Phenylglucuronide, AGE. SEX. GLU, CRE, STE, DIU | 1.94 (1.54 - 2.44) | 2.72∙10^-8^ |
| Pyranose, AGE, SEX, GLU, CRE, APT, RAN | 13.40 (3.80 – 47.18) | 5.30∙10^-5^ |
| Pyruvate, AGE, SEX, GLU, CRE, RAN, AHT | 1.02∙10^-4^ (3.35∙10-6 - 3.13∙10^-3^ | 1.41∙10^-7^ |
| Ser-Hyp*, AGE. SEX. GLU, CRE, RAN, STAT | 5.60∙10^3^ (1.92∙10^2^ - 1.64∙10^5^) | 5.35∙10^-7^ |
| Sphingosine-1-phosphate, AGE, SEX, GLU, CRE, STE, AHT | 7.20∙10^-5^ (1.39∙10^-6^ – 3.71∙10^-3^) | 2.00∙10^-7^ |
| Stearoyl-tyrosine*, AGE. SEX. GLU, CRE, APT, AHT | 1.10∙10^-5^ (1.79∙10^-7^ - 7.02∙10^-4^) | 6.66∙10^-8^ |
| Suberylglycine, AGE. SEX. GLU, CRE, STE, STAT | 179.24 (0 – 1.79∙10^308^) | 0.996 |
| Succinylacetoacetate*, AGE, SEX, GLU, CRE, RAN, AHT | 1.3310^-17^ (0 - 0.52) | 4.62∙10^-2^ |
| Trp-Phe, AGE, SEX, GLU, CRE, RAN | 8.70E∙10^4^ (1.51∙10^3^ - 4.99∙10^6^) | 3.72∙10^-8^ |
| Ursodeoxycholic acid 3-sulfate, AGE, SEX, GLU, CRE, STAT, RAN | 6.92 (2.21 – 21.69) | 9.11∙10^-4^ |
| Ursodeoxycholic acid, AGE, SEX, GLU, CRE, STAT | 1.94 (1.55 – 2.41) | 4.58∙10^-9^ |
| Vaccenyl/Elaidic carnitine, AGE, SEX, GLU, CRE, RAN | 1.88∙10^-3^ (1.44∙10^-4^ - 0.03) | 2.00∙10^-6^ |

CI = Confidence Interval; GLU = Glycaemia; CRE = Creatinine; RAN = Ranitidine; STAT = Statins; STE = Steroids; AHT = Antihypertensive; APT = Antiplatelet; PPI = Proton Pump inhibitor; DIU = Diuretic; APAP = Paracetamol; NSAIDs = Non-steroidal anti-inflammatory drug.

**Table S5.** Metabolites candidates for targeted validation.

| Metabolite | Confidence level | Analytical platform | % change | AUC | Model | |
| --- | --- | --- | --- | --- | --- | --- |
|  |  |  |  |  | **exp (B)** | **P-value** |
| Arginine | 2 | CE-MS/LC-MS(+) | 243 | 0.982 | 2.70E+07 | 4.00E-06 |
| Biliverdin | 3 | LC-MS(-) | 300 | 0.962 | 3.27E+05 | 4.25E-07 |
| Linoleoylcarnitine | 3 | LC-MS(+) | -59 | 0.937 | 4.00E-06 | 4.80E-08 |
| 5-Hydroxyindoleacetic acid | 2 | LC-MS(+) | Cancer group | 0.891 | 3.83E+00 | 7.00E-06 |
|  |  |  |  |  |  |  |
|  |  |  |  |  |  |  |
| Hydroxydodecanoic acid | 2 | LC-MS(-) | Cancer group | 0.887 | 3.04E+00 | 3.07E-12 |
|  |  |  |  |  |  |  |
| Bilirubin | 2 | LC-MS(-)/LC-MS(+) | 674 | 0.861 | 2.30E+00 | 2.21E-10 |
| Hydroxydodecanedioic acid | 2 | LC-MS(-) | Cancer group | 0.876 | 2.31E+00 | 8.84E-12 |
| 1-Methyladenosine | 2 | CE-MS | 41 | 0.857 | 2.63E+07 | 1.49E-07 |
| HETE | 2 | LC-MS(-) | Cancer group | 0.846 | 2.00E+00 | 2.22E-09 |
| Sphingosine-1-phosphate | 2 | LC-MS(+) | -30 | 0.838 | 7.20E-05 | 2.00E-06 |
| Oleoylcarnitine | 2 | LC-MS(+) | -41 | 0.828 | 1.88E-03 | 2.00E-06 |
| Ursodeoxycholic acid 3-sulfate | 3 | LC-MS(-) | 130 | 0.807 | 6.92E+00 | 9.11E-04 |
| Ursodeoxycholic acid | 2 | LC-MS(-) | 188 | 0.779 | 1.94E+00 | 4.58E-09 |

**Table S6. Validation of potential diagnostic biomarkers of NETs.** This table summarizes the experimental data from the LC-QQQ-MS/MS analysis of the 13 differential metabolites (out of 48) between NET (N=77) and non-cancer (N=68) patients. RT, retention time and the transition data used for the quantification of each metabolite, as well as the concentration for each group, the p value and the % change are shown for each metabolite. Overall, 10 metabolites were validated as diagnostic biomarkers of NETs.

| Compound | Formula Molecular | RT^b^ (min) | Transition | Polarity | *p* value | % change | Concentration NETs  (ng mL^‐1^) | Concentration non-cancer  (ng mL^‐1^) | Calibration curve | r^2^ |
| --- | --- | --- | --- | --- | --- | --- | --- | --- | --- | --- |
| Arginine | C6H14N4O2 | 0.521 | 175.1 -> 70.1 | + | 9.67E-07 | 341 | 268 ± 76 | 60.8 ± 6.8 | y = 145.7x + 48.9 | 0.9993 |
| 1-Methyladenosine | C11H15N5O4 | 0.602 | 282.1 -> 150.0 | + | 1.01E-19 | 24 | 6.73 ± 0.18 | 5.44 ± 0.16 | y = 2228.6x - 1063.7 | 0.9991 |
| Bilirubin | C33H36N4O6 | 4.162 | 585.3 -> 299.1 | + | NP | NP | NP | NP | y = 25.801x + 526.49 | 0.96 |
| Biliverdin | C33H34N4O6 | 2.376 | 583.3 -> 297.1 | + | 5.12E-14 | 179 | 97 ± 12 | 34.8 ± 5.6 | y = 8.6391x + 2.013 | 0.999 |
| 5-Hydroxyindoleacetic acid | C10H9NO3 | 1.540 | 190.1 -> 144.0 | - | 1.40E-06 | 407 | 152 ± 44 | 30.0 ± 8.0 | y = 2.1188x - 9.8133 | 0.9998 |
| 3-Hydroxydodecanedioic acid | C12H22O5 | 1.746 | 245.1 -> 185.2 | - | NP | NP | NP | NP | y = 30.17635x + 0.23980 | 0.994 |
| Linoleoylcarnitine | C25H45NO4 | 2.529 | 424.3 -> 85.0 | + | 2.00E-16 | -53 | 81.2 ± 7.6 | 173 ± 16 | y = 784.09x - 3404.6 | 0.9999 |
| Oleoylcarnitine | C25H47NO4 | 2.570 | 426.4 -> 85.0 | + | 1.07E-09 | -50 | 120 ± 15 | 238 ± 31 | y = 1003.7x - 6622.4 | 0.9998 |
| Sphingosine-1-phosphate | C18H38NO5P | 2.773 | 380.3 -> 82.1 | + | 4.70E-13 | -27 | 353 ± 25 | 482 ± 18 | y = 107.95x - 1072.3 | 0.9994 |
| 3-Hydroxydodecanoic acid | C12H24O3 | 2.778 | 215.2 -> 59.1 | - | NP | NP | NP | NP | y = 70.765x + 120.68 | 0.9999 |
| HETE | C20H32O3 | 3.018 | 319.2 -> 301.2 | - | 2.13E-02 | 1319 | 21 ± 16 | 1.48 ± 0.64 | y = 25.441x | 0.9997 |
| Ursodeoxycholic acid | C24H40O4 | 2.583 | 391.2 -> 391.2 | - | 4.42E-02 | 90 | 91 ± 36 | 48 ± 18 | y = 117.01x + 837.82 | 0.9998 |
| Ursodeoxycholic acid 3-sulfate | C24H40O7S | 2.448 | 475.1 -> 475.1 | - | 1.86E-05 | 27 | 33.2 ± 2.3 | 26.2 ± 2.0 | y = 129.62x - 342.57 | 0.9999 |

**Table S7.** Relevant metabolic pathways related to the identified differential plasma metabolites in NET patients by Metabolite Pathway Analysis (MPA). This table summarizes the enriched metabolic signalling pathways (*FDR < 0.05*) related to the set of identified differential metabolites (N= 155) in the plasma of NET patients (n=77), when compared to non-cancer individuals (N=68), obtained through MPA in *MetaboAnalyst 4* platform. The number of identified metabolites which belong to each pathway is indicated in the column *hits,* and the total number of metabolites that compose each metabolic pathway in the column *total pathway*. The raw p-value (*GlobalTest)* and the adjusted p-value by multiple analysis (*Bonferroni-Holm* y *FDR*) were calculated for each metabolic pathway. The Impact column estimates the node (metabolites) importance in the pathway. Overall, 32 enriched metabolic pathways were identified and are depicted in the table by decreasing order of impact (*Imp*) of the metabolites included in the topological analysis.

| **Pathway** | **Total Pathway** | **Hits** | **Raw p** | **-log(p)** | **Holm adjust** | **FDR** | **Impact** |
| --- | --- | --- | --- | --- | --- | --- | --- |
| Starch and sucrose metabolism | 18 | 1 | 1,90E-07 | 15,476 | 3,1788E-06 | 2,9636E-07 | 0,4207 |
| Alanine, aspartate and glutamate metabolism | 28 | 4 | 5,88E-49 | 111,05 | 2,2363E-47 | 1,1476E-47 | 0,33734 |
| Arachidonic acid metabolism | 36 | 2 | 3,76E-03 | 5,5838 | 0,041342 | 0,0050544 | 0,3135 |
| Sphingolipid metabolism | 21 | 3 | 4,18E-16 | 35,411 | 1,0872E-14 | 1,1648E-15 | 0,29412 |
| Pyruvate metabolism | 22 | 2 | 1,54E-35 | 80,159 | 5,0802E-34 | 8,577E-35 | 0,29082 |
| Glycine, serine and threonine metabolism | 33 | 2 | 3,69E-19 | 42,443 | 9,9663E-18 | 1,1074E-18 | 0,24577 |
| Citrate cycle (TCA cycle) | 20 | 4 | 3,32E-34 | 77,087 | 1,0634E-32 | 1,6201E-33 | 0,23173 |
| Glycerophospholipid metabolism | 36 | 3 | 1,87E-07 | 15,492 | 3,1788E-06 | 2,9636E-07 | 0,21631 |
| Arginine and proline metabolism | 38 | 3 | 1,72E-44 | 100,77 | 6,3629E-43 | 2,2356E-43 | 0,16849 |
| Glutathione metabolism | 28 | 4 | 8,02E-40 | 90,021 | 2,8086E-38 | 6,2591E-39 | 0,16845 |
| Glyoxylate and dicarboxylate metabolism | 32 | 6 | 5,17E-40 | 90,46 | 1,8621E-38 | 5,0432E-39 | 0,16138 |
| Pentose and glucuronate interconversions | 18 | 1 | 1,72E-03 | 6,3682 | 0,024012 | 0,0025727 | 0,14062 |
| Arginine biosynthesis | 14 | 4 | 2,60E-50 | 114,17 | 1,0143E-48 | 1,0143E-48 | 0,13705 |
| Porphyrin and chlorophyll metabolism | 30 | 3 | 1,05E-20 | 46,006 | 2,9317E-19 | 3,4029E-20 | 0,12753 |
| Histidine metabolism | 16 | 2 | 2,51E-13 | 29,015 | 5,5145E-12 | 5,431E-13 | 0,12295 |
| Glycolysis / Gluconeogenesis | 26 | 1 | 8,16E-21 | 46,255 | 2,5292E-19 | 2,8926E-20 | 0,10044 |
| Galactose metabolism | 27 | 3 | 1,65E-07 | 15,615 | 2,9779E-06 | 2,9328E-07 | 0,08787 |
| Tryptophan metabolism | 41 | 4 | 2,34E-09 | 19,875 | 4,9053E-08 | 4,7946E-09 | 0,04081 |
| Purine metabolism | 65 | 2 | 1,95E-03 | 6,2402 | 0,025342 | 0,0028158 | 0,01651 |
| Glycosylphosphatidylinositol (GPI)-anchor biosynthesis | 14 | 1 | 1,87E-08 | 17,793 | 3,7449E-07 | 3,6513E-08 | 0,00399 |
| Aminoacyl-tRNA biosynthesis | 48 | 5 | 3,47E-39 | 88,558 | 1,1784E-37 | 2,2528E-38 | 0 |
| Cysteine and methionine metabolism | 33 | 1 | 8,16E-21 | 46,255 | 2,5292E-19 | 2,8926E-20 | 0 |
| Tyrosine metabolism | 42 | 1 | 8,16E-21 | 46,255 | 2,5292E-19 | 2,8926E-20 | 0 |
| β-Alanine metabolism | 21 | 1 | 9,93E-15 | 32,243 | 2,4829E-13 | 2,2784E-14 | 0 |
| Nicotinate and nicotinamide metabolism | 15 | 1 | 9,93E-15 | 32,243 | 2,4829E-13 | 2,2784E-14 | 0 |
| Pantothenate and CoA biosynthesis | 19 | 1 | 9,93E-15 | 32,243 | 2,4829E-13 | 2,2784E-14 | 0 |
| Amino sugar and nucleotide sugar metabolism | 37 | 1 | 2,73E-08 | 17,417 | 5,1851E-07 | 5,0682E-08 | 0 |
| Neomycin, kanamycin and gentamicin biosynthesis | 2 | 1 | 1,90E-07 | 15,476 | 3,1788E-06 | 2,9636E-07 | 0 |
| Biosynthesis of unsaturated fatty acids | 36 | 3 | 2,42E-03 | 6,0235 | 0,029055 | 0,0033724 | 0 |
| Pyrimidine metabolism | 39 | 1 | 3,07E-02 | 3,484 | 0,30684 | 0,037397 | 0 |
| D-Glutamine and D-glutamate metabolism | 6 | 1 | 3,07E-02 | 3,484 | 0,30684 | 0,037397 | 0 |
| Nitrogen metabolism | 6 | 1 | 3,07E-02 | 3,484 | 0,30684 | 0,037397 | 0 |

**Table S8.** Relevant metabolic pathways related to the identified differential plasma metabolites in NET patients by Metabolite Set Enrichment Analysis (MSEA). This table summarizes the enriched metabolic signalling pathways (*FDR < 0.05*) related to the selected metabolites (N= 155) in the plasma of NET patients (n=77), when compared to non-cancer individuals (N=68), obtained through MSEA in *MetaboAnalyst 4* platform. The number of identified metabolites that belong to each pathway is indicated in the column *hits,* and the total number of metabolites that compose each pathway in the column *total pathway*. The *Q-statistic* and the expected Q are also shown. The raw p-value and the adjusted p-value by multiple analysis (*Bonferroni-Holm* y *FDR*) were calculated for each metabolic gene set. Overall, 34 significantly enriched metabolic pathways were identified and are ordered in the table by FDR.

| Pathway | Total Pathway | Hits | Q Statistic | Expected Q | Raw p | Holm adjust | FDR |
| --- | --- | --- | --- | --- | --- | --- | --- |
| Aspartate Metabolism | 35 | 3 | 28.809 | 0.69444 | 2.50E-57 | 1.05E-55 | 1.05E-55 |
| Arginine and Proline Metabolism | 53 | 4 | 38.272 | 0.69444 | 3.07E-52 | 1.26E-50 | 6.44E-51 |
| Urea Cycle | 29 | 5 | 40.438 | 0.69444 | 8.85E-51 | 3.54E-49 | 1.24E-49 |
| Glycolysis | 25 | 2 | 31.619 | 0.69444 | 6.25E-48 | 2.44E-46 | 4.38E-47 |
| Glucose-Alanine Cycle | 13 | 2 | 31.619 | 0.69444 | 6.25E-48 | 2.44E-46 | 4.38E-47 |
| Gluconeogenesis | 35 | 2 | 31.619 | 0.69444 | 6.25E-48 | 2.44E-46 | 4.38E-47 |
| Glycine and Serine Metabolism | 59 | 4 | 41.168 | 0.69444 | 2.47E-44 | 8.90E-43 | 1.48E-43 |
| Spermidine and Spermine Biosynthesis | 18 | 1 | 69.861 | 0.69444 | 4.55E-39 | 1.59E-37 | 2.39E-38 |
| Pyruvate Metabolism | 48 | 2 | 59.344 | 0.69444 | 1.54E-35 | 5.23E-34 | 6.47E-35 |
| Pyruvaldehyde Degradation | 10 | 2 | 59.344 | 0.69444 | 1.54E-35 | 5.23E-34 | 6.47E-35 |
| Citric Acid Cycle | 32 | 4 | 35.905 | 0.69444 | 3.32E-34 | 1.06E-32 | 1.27E-33 |
| Sphingolipid Metabolism | 40 | 3 | 23.905 | 0.69444 | 2.71E-32 | 8.39E-31 | 9.47E-32 |
| Ammonia Recycling | 32 | 4 | 20.868 | 0.69444 | 5.19E-31 | 1.56E-29 | 1.56E-30 |
| Glutamate Metabolism | 49 | 4 | 20.868 | 0.69444 | 5.19E-31 | 1.56E-29 | 1.56E-30 |
| Glutathione Metabolism | 21 | 3 | 21.78 | 0.69444 | 9.13E-26 | 2.56E-24 | 2.56E-25 |
| Transfer of Acetyl Groups into Mitochondria | 22 | 3 | 22.487 | 0.69444 | 1.61E-25 | 4.34E-24 | 4.22E-25 |
| Cysteine Metabolism | 26 | 1 | 45.902 | 0.69444 | 8.16E-21 | 2.12E-19 | 2.02E-20 |
| Alanine Metabolism | 17 | 2 | 22.964 | 0.69444 | 3.69E-19 | 9.23E-18 | 8.61E-19 |
| Amino Sugar Metabolism | 33 | 2 | 24.563 | 0.69444 | 9.93E-18 | 2.38E-16 | 2.19E-17 |
| Porphyrin Metabolism | 40 | 3 | 19.779 | 0.69444 | 2.08E-16 | 4.77E-15 | 4.36E-16 |
| Warburg Effect | 58 | 5 | 14.982 | 0.69444 | 1.95E-15 | 4.29E-14 | 3.90E-15 |
| Tyrosine Metabolism | 72 | 1 | 34.319 | 0.69444 | 9.93E-15 | 2.09E-13 | 1.74E-14 |
| Beta-Alanine Metabolism | 34 | 1 | 34.319 | 0.69444 | 9.93E-15 | 2.09E-13 | 1.74E-14 |
| Malate-Aspartate Shuttle | 10 | 1 | 34.319 | 0.69444 | 9.93E-15 | 2.09E-13 | 1.74E-14 |
| Purine Metabolism | 74 | 4 | 10.69 | 0.69444 | 7.08E-13 | 1.27E-11 | 1.19E-12 |
| Tryptophan Metabolism | 60 | 4 | 10.135 | 0.69444 | 2.34E-09 | 3.97E-08 | 3.77E-09 |
| Lactose Synthesis | 20 | 1 | 17.336 | 0.69444 | 1.90E-07 | 3.04E-06 | 2.85E-07 |
| Lactose Degradation | 9 | 1 | 17.336 | 0.69444 | 1.90E-07 | 3.04E-06 | 2.85E-07 |
| Galactose Metabolism | 38 | 2 | 8.8226 | 0.69444 | 4.54E-06 | 6.36E-05 | 6.58E-06 |
| Alpha Linolenic Acid and Linoleic Acid Metabolism | 19 | 3 | 6.0314 | 0.69444 | 0.00015607 | 0.0020289 | 0.0002185 |
| Arachidonic Acid Metabolism | 69 | 1 | 4.9694 | 0.69444 | 0.0070369 | 0.084442 | 0.0095338 |
| Methionine Metabolism | 43 | 2 | 2.5649 | 0.69444 | 0.02382 | 0.26203 | 0.031264 |
| Nicotinate and Nicotinamide Metabolism | 37 | 1 | 3.2244 | 0.69444 | 0.030684 | 0.30684 | 0.037904 |
| Phenylacetate Metabolism | 9 | 1 | 3.2244 | 0.69444 | 0.030684 | 0.30684 | 0.037904 |
| Pyrimidine Metabolism | 59 | 2 | 2.1157 | 0.69444 | 0.046438 | 0.37151 | 0.055726 |
| Bile Acid Biosynthesis | 65 | 2 | 1.8928 | 0.69444 | 0.064894 | 0.45426 | 0.075709 |
| Phenylalanine and Tyrosine Metabolism | 28 | 1 | 1.6765 | 0.69444 | 0.12061 | 0.72369 | 0.13691 |
| Phospholipid Biosynthesis | 29 | 1 | 0.61779 | 0.69444 | 0.34736 | 1 | 0.38392 |
| Lysine Degradation | 30 | 1 | 0.38391 | 0.69444 | 0.45908 | 1 | 0.4944 |
| Glycerolipid Metabolism | 25 | 1 | 0.30878 | 0.69444 | 0.50679 | 1 | 0.53213 |
| Retinol Metabolism | 37 | 1 | 0.16657 | 0.69444 | 0.62597 | 1 | 0.64124 |
| Carnitine Synthesis | 22 | 1 | 0.025872 | 0.69444 | 0.84772 | 1 | 0.84772 |

**Table S9.** Relevant metabolic pathways related to the diagnostic biomarker metabolites in NET patients by Metabolite Pathway Analysis (MPA). This table summarizes the enriched metabolic signalling pathways (*FDR < 0.05*) related to the selected metabolites (N= 48) in the plasma of NET patients (n=77), when compared to non-cancer individuals (N=68), obtained through MPA in *MetaboAnalyst 4* platform. The number of identified metabolites that belong to each pathway is indicated in the column *hits,* and the total number of metabolites that compose each metabolic pathway in the column *total pathway*. The raw p-value (*GlobalTest)* and the adjusted p-value by multiple analysis (*Bonferroni-Holm* y *FDR*) were calculated for each metabolic pathway. The Impact column estimates the node (metabolite) importance in the pathway. Overall, 23 enriched metabolic pathways were identified and have been ordered in the table by the impact (*Imp*) of the metabolites included in the topological analysis.

| Pathway | Total Pathway | Hits | Raw p | -log(p) | Holm adjust | FDR | Impact |
| --- | --- | --- | --- | --- | --- | --- | --- |
| Starch and sucrose metabolism | 18 | 1 | 2.11E-05 | 4.6763 | 4.21E-05 | 2.11E-05 | 0.4207 |
| Pyruvate metabolism | 22 | 2 | 7.22E-37 | 36.141 | 1.23E-35 | 2.37E-36 | 0.29082 |
| Alanine, aspartate and glutamate metabolism | 28 | 2 | 4.30E-31 | 30.367 | 6.88E-30 | 1.24E-30 | 0.22356 |
| Arginine and proline metabolism | 38 | 3 | 2.01E-43 | 42.698 | 3.81E-42 | 9.23E-43 | 0.16849 |
| Arginine biosynthesis | 14 | 3 | 1.99E-52 | 51.7 | 4.19E-51 | 1.53E-51 | 0.13705 |
| Porphyrin and chlorophyll metabolism | 30 | 2 | 6.78E-13 | 12.169 | 4.07E-12 | 8.67E-13 | 0.12753 |
| Glycolysis / Gluconeogenesis | 26 | 1 | 1.02E-21 | 20.99 | 1.43E-20 | 1.81E-21 | 0.10044 |
| Citrate cycle (TCA cycle) | 20 | 2 | 3.30E-56 | 55.481 | 7.60E-55 | 3.80E-55 | 0.09637 |
| Galactose metabolism | 27 | 2 | 8.68E-06 | 5.0616 | 2.60E-05 | 9.50E-06 | 0.08787 |
| Tryptophan metabolism | 41 | 2 | 8.01E-12 | 11.096 | 4.01E-11 | 9.70E-12 | 0.04081 |
| Sphingolipid metabolism | 21 | 1 | 1.97E-27 | 26.706 | 2.95E-26 | 5.03E-27 | 0.02434 |
| Glyoxylate and dicarboxylate metabolism | 32 | 2 | 3.30E-56 | 55.481 | 7.60E-55 | 3.80E-55 | 0.02381 |
| Glutathione metabolism | 28 | 2 | 2.67E-41 | 40.573 | 4.81E-40 | 1.02E-40 | 0.02292 |
| Aminoacyl-tRNA biosynthesis | 48 | 2 | 1.30E-45 | 44.887 | 2.60E-44 | 7.46E-45 | 0 |
| Glycine, serine and threonine metabolism | 33 | 1 | 1.02E-21 | 20.99 | 1.43E-20 | 1.81E-21 | 0 |
| Cysteine and methionine metabolism | 33 | 1 | 1.02E-21 | 20.99 | 1.43E-20 | 1.81E-21 | 0 |
| Tyrosine metabolism | 42 | 1 | 1.02E-21 | 20.99 | 1.43E-20 | 1.81E-21 | 0 |
| Histidine metabolism | 16 | 1 | 9.17E-17 | 16.038 | 9.17E-16 | 1.24E-16 | 0 |
| beta-Alanine metabolism | 21 | 1 | 9.17E-17 | 16.038 | 9.17E-16 | 1.24E-16 | 0 |
| Nicotinate and nicotinamide metabolism | 15 | 1 | 9.17E-17 | 16.038 | 9.17E-16 | 1.24E-16 | 0 |
| Pantothenate and CoA biosynthesis | 19 | 1 | 9.17E-17 | 16.038 | 9.17E-16 | 1.24E-16 | 0 |
| Amino sugar and nucleotide sugar metabolism | 37 | 1 | 3.94E-06 | 5.4042 | 1.58E-05 | 4.53E-06 | 0 |
| Neomycin, kanamycin and gentamicin biosynthesis | 2 | 1 | 2.11E-05 | 4.6763 | 4.21E-05 | 2.11E-05 | 0 |

**Table S10.** Relevant metabolic pathways related to the diagnostic biomarker metabolites in NET patients by Metabolite Set Enrichment Analysis (MSEA). This table summarizes the enriched metabolic signalling pathways (*FDR < 0.05*) related to the selected metabolites (N= 48) in the plasma of NET patients (n=77), when compared to non-cancer individuals (N=68), obtained through MSEA in *MetaboAnalyst 4* platform. The number of identified metabolites that belong to each pathway is indicated in the column *hits,* and the total number of metabolites that compose each metabolic pathway in the column *total pathway*. The *Q-statistic* and the expected Q are also shown. The raw p-value and the adjusted p-value by multiple analysis (*Bonferroni-Holm* y *FDR*) were calculated for each metabolic gene set. Overall, 30 significantly enriched metabolic pathways were identified and have been ordered in the table by FDR.

| Pathway | Total Pathway | Hits | Q Statistic | Expected Q | Raw p | Holm adjust | FDR |
| --- | --- | --- | --- | --- | --- | --- | --- |
| Citric Acid Cycle | 32 | 2 | 68.138 | 0.69444 | 3.30E-56 | 9.91E-55 | 9.91E-55 |
| Arginine and Proline Metabolism | 53 | 3 | 47.931 | 0.69444 | 1.99E-52 | 5.78E-51 | 2.99E-51 |
| Urea Cycle | 29 | 4 | 47.808 | 0.69444 | 7.29E-52 | 2.04E-50 | 7.29E-51 |
| Aspartate Metabolism | 35 | 2 | 38.216 | 0.69444 | 1.30E-45 | 3.51E-44 | 9.74E-45 |
| Glycine and Serine Metabolism | 59 | 3 | 50.931 | 0.69444 | 2.01E-43 | 5.22E-42 | 1.20E-42 |
| Pyruvate Metabolism | 48 | 2 | 61.053 | 0.69444 | 7.22E-37 | 1.81E-35 | 3.09E-36 |
| Pyruvaldehyde Degradation | 10 | 2 | 61.053 | 0.69444 | 7.22E-37 | 1.81E-35 | 3.09E-36 |
| Spermidine and Spermine Biosynthesis | 18 | 1 | 67.36 | 0.69444 | 1.38E-36 | 3.18E-35 | 5.19E-36 |
| Glycolysis | 25 | 2 | 29.68 | 0.69444 | 1.32E-35 | 2.91E-34 | 3.06E-35 |
| Glucose-Alanine Cycle | 13 | 2 | 29.68 | 0.69444 | 1.32E-35 | 2.91E-34 | 3.06E-35 |
| Gluconeogenesis | 35 | 2 | 29.68 | 0.69444 | 1.32E-35 | 2.91E-34 | 3.06E-35 |
| Transfer of Acetyl Groups into Mitochondria | 22 | 2 | 29.68 | 0.69444 | 1.32E-35 | 2.91E-34 | 3.06E-35 |
| Warburg Effect | 58 | 2 | 29.68 | 0.69444 | 1.32E-35 | 2.91E-34 | 3.06E-35 |
| Ammonia Recycling | 32 | 2 | 42.939 | 0.69444 | 4.30E-31 | 7.31E-30 | 8.60E-31 |
| Glutamate Metabolism | 49 | 2 | 42.939 | 0.69444 | 4.30E-31 | 7.31E-30 | 8.60E-31 |
| Sphingolipid Metabolism | 40 | 2 | 34.073 | 0.69444 | 3.97E-22 | 5.96E-21 | 7.45E-22 |
| Cysteine Metabolism | 26 | 1 | 47.439 | 0.69444 | 1.02E-21 | 1.43E-20 | 1.61E-21 |
| Amino Sugar Metabolism | 33 | 1 | 47.439 | 0.69444 | 1.02E-21 | 1.43E-20 | 1.61E-21 |
| Alanine Metabolism | 17 | 1 | 47.439 | 0.69444 | 1.02E-21 | 1.43E-20 | 1.61E-21 |
| Tyrosine Metabolism | 72 | 1 | 38.438 | 0.69444 | 9.17E-17 | 1.01E-15 | 1.20E-16 |
| Beta-Alanine Metabolism | 34 | 1 | 38.438 | 0.69444 | 9.17E-17 | 1.01E-15 | 1.20E-16 |
| Purine Metabolism | 74 | 1 | 38.438 | 0.69444 | 9.17E-17 | 1.01E-15 | 1.20E-16 |
| Malate-Aspartate Shuttle | 10 | 1 | 38.438 | 0.69444 | 9.17E-17 | 1.01E-15 | 1.20E-16 |
| Porphyrin Metabolism | 40 | 2 | 22.444 | 0.69444 | 6.78E-13 | 4.75E-12 | 8.48E-13 |
| Tryptophan Metabolism | 60 | 2 | 15.547 | 0.69444 | 8.01E-12 | 4.81E-11 | 9.62E-12 |
| Galactose Metabolism | 38 | 1 | 11.921 | 0.69444 | 2.11E-05 | 0.00010537 | 2.26E-05 |
| Lactose Synthesis | 20 | 1 | 11.921 | 0.69444 | 2.11E-05 | 0.00010537 | 2.26E-05 |
| Lactose Degradation | 9 | 1 | 11.921 | 0.69444 | 2.11E-05 | 0.00010537 | 2.26E-05 |
| Glutathione Metabolism | 21 | 1 | 10.614 | 0.69444 | 6.37E-05 | 0.0001274 | 6.59E-05 |
| Alpha Linolenic Acid and Linoleic Acid Metabolism | 19 | 1 | 4.7442 | 0.69444 | 0.0084941 | 0.0084941 | 0.0084941 |

**Table S11.** Distribution of age, gender and body mass index in NET patients and non-cancer individuals.

|  | Neuroendocrine Tumors  N= 77 (100%) | Non-cancer Individuals  N= 68 (100%) |
| --- | --- | --- |
| **Gender (N (%))** |  |  |
| Female | 35 (45.5%) | 41 (60.3%) |
| Male | 42 (54.5%) | 27 (39.7%) |
| **Age (years)** |  |  |
| Median value (range) | 63 (37-83) | 61.7 ( 38-83) |
| **BMI** |  |  |
| Median value (range) | 25.9 (17.2- 52.5) | 26.8 (20.7 – 35.9) |

BMI: Body Mass Index.

**Table S12.** Standards and the corresponding sources used for the targeted analysis.

| **Metabolite** | **Brand** | **Company** | **Purity** |
| --- | --- | --- | --- |
| Arginine | SIGMA ALDRICH | Merck Life Science S.L. Calle Maria de Molina 40 E-28006 MADRID | >98% |
| Biliverdin | SIGMA ALDRICH | Merck Life Science S.L. Calle Maria de Molina 40 E-28006 MADRID | ≥97% |
| Linoleoylcarnitine | SIGMA ALDRICH | Merck Life Science S.L. Calle Maria de Molina 40 E-28006 MADRID | ≥95% |
| 5-Hydroxyindoleacetic acid | Acros Organics | Fisher Scientific One Reagent Lane Fair Lawn, NJ 07410 | ≥99% |
| 3-Hydroxydodecanoic acid | SIGMA ALDRICH | Merck Life Science S.L. Calle Maria de Molina 40 E-28006 MADRID | ≥99% |
| Bilirubin | SIGMA ALDRICH | Merck Life Science S.L. Calle Maria de Molina 40 E-28006 MADRID | ≥95% |
| 1-Methyladenosine | Cayman Chemical Company | Cayman Chemical Company 1180 E. Ellsworth Rd. Ann Arbor, MI 48108 | ≥98% |
| 15-HETE | Avanti Polar Lipids, INC | 700 Industrial Park Drive Alabaster, Al 35007 United States of America | ≥99% |
| Sphingosine-1-phosphate | SIGMA ALDRICH | Merck Life Science S.L. Calle Maria de Molina 40 E-28006 MADRID | ≥98% |
| 3-Hydroxydodecanedioic acid | Toronto Research Chemicals | Toronto Research Chemicals 2 Brisbane Road Toronto, ON M3J 2J8 CANADA | 93% |
| Ursodeoxycholic acid 3-sulfate | SIGMA ALDRICH | Merck Life Science S.L. Calle Maria de Molina 40 E-28006 MADRID | ≥98% |
| Oleoylcarnitine | Larodan AB | Larodan AB Karolinska Institutet Science Park Retzius väg 8 SE-171 65 SOLNA Sweden | ≥97% |
| Ursodeoxycholic acid | SIGMA ALDRICH | Merck Life Science S.L. Calle Maria de Molina 40 E-28006 MADRID | ≥99% |

**Table S13.** Drug intake of NET and non-cancer patients.

| **Drug class** | **NET patients N (%)** | **Non-cancer N (%)** |
| --- | --- | --- |
| Alpha-adrenergic Blockers | 2 (2.56%) | 2 (2.9%) |
| Analgesics | 20 (25.97%) | 11 (16.2%) |
| Anaesthetics | 1 (1.28%) | 0 (0%) |
| Anticholinergics | 2 (2.56%) | 0 (0%) |
| Anticoagulants/Antiaggregants | 12 (15.58%) | 2 (2.9%) |
| Antidiabetics | 5 (6.49%) | 5 (7.4%) |
| Antidiarrheals | 4 (5.13%) | 0 (0%) |
| Antifungals | 1 (1.28%) | 0 (0%) |
| Antigout agents | 3 (3.85%) | 1 (1.4%) |
| Antihistaminics | 3 (3.85%) | 2 (2.9%) |
| Antihypertensive agents | 42 (54.54%) | 20 (29.4%) |
| Antivirals | 2 (2.56%) | 0 (0%) |
| Bile Acids Sequestrants | 1 (1.28%) | 1 (1.4%) |
| Bronchodilators | 7 (8.97%) | 4 (5.8%) |
| Diuretics | 14 (17.95%) | 4 (5.8%) |
| H2 Receptor Blockers | 8 (10.26%) | 0 (0%) |
| Laxative agents | 2 (2.56%) | 0 (0%) |
| Lipid-Lowering Medications | 16 (20.77%) | 19 (27.9%) |
| Medicinal Herbs | 1 (1.28%) | 0 (0%) |
| Nitrates | 1 (1.28%) | 0 (0%) |
| Propulsives | 2 (2.56%) | 0 (0%) |
| Proton Pump Inhibitors | 15 (19.23%) | 9 (13.2%) |
| Psychoactive drugs | 19 (24.67%) | 11 (16.2%) |
| Steroids | 4 (5.13%) | 0 (0%) |
| Thyroid agents | 6 (7.79%) | 6 (8.8%) |
| Vitamins and dietary supplements | 24 (31.16%) | 4 (5.8%) |
